# Structure of a functional cap-binding domain in Rift Valley fever virus L protein

**DOI:** 10.1371/journal.ppat.1007829

**Published:** 2019-05-28

**Authors:** Nadja Gogrefe, Sophia Reindl, Stephan Günther, Maria Rosenthal

**Affiliations:** 1 Department of Virology, Bernhard Nocht Institute for Tropical Medicine, Hamburg, Germany; 2 German Center for Infection Research (DZIF), Partner site Hamburg–Lübeck–Borstel–Riems, Germany; Oxford University, UNITED KINGDOM

## Abstract

Rift Valley fever virus (RVFV) belongs to the family of *Phenuiviridae* within the order of *Bunyavirales*. The virus may cause fatal disease both in livestock and humans, and therefore, is of great economical and public health relevance. In analogy to the influenza virus polymerase complex, the bunyavirus L protein is assumed to bind to and cleave off cap structures of cellular mRNAs to prime viral transcription. However, even though the presence of an endonuclease in the N-terminal domain of the L protein has been demonstrated for several bunyaviruses, there is no evidence for a cap-binding site within the L protein. We solved the structure of a C-terminal 117 amino acid-long domain of the RVFV L protein by X-ray crystallography. The overall fold of the domain shows high similarity to influenza virus PB2 cap-binding domain and the putative non-functional cap-binding domain of reptarenaviruses. Upon co-crystallization with m^7^GTP, we detected the cap-analogue bound between two aromatic side chains as it has been described for other cap-binding proteins. We observed weak but specific interaction with m^7^GTP rather than GTP *in vitro* using isothermal titration calorimetry. The importance of m^7^GTP-binding residues for viral transcription was validated using a RVFV minigenome system. In summary, we provide structural and functional evidence for a cap-binding site located within the L protein of a virus from the *Bunyavirales* order.

## Author summary

Rift Valley fever virus (RVFV) is endemic to sub-Saharan Africa and the Arabian Peninsula and leads to abortions in and death of ruminants. The virus can also be transmitted to humans causing febrile illness up to hemorrhagic fever with the possibility of fatal outcome. As there is currently no human vaccine or specific treatment available and because of the high epidemic potential, WHO has listed RVFV on its R&D Blueprint for urgent development of medical countermeasures. In order to amplify, the virus needs to transcribe and replicate the viral genome inside the cell cytoplasm. For transcription, the virus uses a process called cap-snatching, which is essentially depending on two functions presumed to reside within the large viral L protein: the ability to bind cap-structures and the activity of cleaving them off from cellular mRNA. Both functions could serve as specific targets for antiviral drug design. We identified and solved the structure of the cap-binding domain of RVFV and provide the first evidence for the presence of a functional cap-binding site in the L protein of bunyaviruses. Comparison with cap-binding proteins of related viruses revealed similarities and important differences critical for the development of potential broad-spectrum antivirals.

## Introduction

Rift Valley fever virus (RVFV) belongs to the family of *Phenuiviridae* within the order of *Bunyavirales* with a single stranded RNA genome in negative orientation (https://talk.ictvonline.org/taxonomy/). RVFV is endemic to sub-Saharan African countries but has also spread to the Arabian Peninsula. The virus infects ruminants and pseudoruminants leading to abortions in pregnant animals and high mortality among young animals. The virus can also be transmitted to humans causing febrile illness with the possibility for severe disease even with fatal outcome [1, 2]. Due to the high economical burden of RVFV outbreaks among livestock, the possibility of severe human disease without effective antiviral treatment options and the epidemic potential RVFV is listed on the WHO R&D Blueprint. The WHO urges to focus R&D on this pathogen to develop medical countermeasures [3].

All viruses from the order of *Bunyavirales* amplify in the cell cytoplasm and the key factor for viral replication is the large L protein (220–450 kDa). The L protein is a multifunctional enzyme catalyzing genome replication as well as viral transcription. Whereas genome replication is initiated *de novo* by a prime-and-realign mechanism [4–7], viral transcription is a primer-dependent process. Messenger RNAs of most segmented negative sense RNA viruses, including bunyaviruses, contain additional non-templated host-derived sequences at their 5' ends. Therefore, cap-snatching was proposed to be a common mechanism in these viruses to initiate viral transcription [5, 6, 8–10]. As demonstrated for the influenza virus polymerase complex [11], the bunyavirus L protein is assumed to bind to cap structures of cellular mRNAs, while an endonuclease cleaves the mRNA some nucleotides downstream of the cap creating a short RNA fragment. This capped RNA fragment is subsequently used to prime viral transcription. The length of these sequences differs between the virus families [6, 8–10, 12–15]. This hypothesis is strengthened by the identification and biochemical characterization of an endonuclease in the N-terminus of the L protein for several viruses of the *Bunyavirales* order [7, 16–21]. However, there is no convincing evidence for a cap-binding site within the L protein so far. Mutational studies on the Lassa virus and RVFV L proteins using minireplicon systems revealed a specific role of the C-terminus in viral transcription [22, 23]. A crystal structure of a C-terminal L protein fragment from a reptarenavirus showed high structural similarity to influenza virus PB2 cap-binding domain but the reptarenavirus domain was lacking the expected cap-binding site arrangement of two aromatic side chains as well as any *in vitro* cap-binding activity [21]. The structure of the La Crosse virus L protein, published in 2015 by Gerlach *et al*. [24], is missing the C-terminal region, allowing only for the conclusion that there is no cap-binding domain present in any other part of the L protein.

We solved the structure of a C-terminal domain of the RVFV L protein and demonstrated specific binding to a cap-structure. We validated the importance of several specific residues implicated in cap-binding for viral transcription using a RVFV minigenome system. Furthermore, we compared the new structure with the structures of influenza virus cap-binding domain as well as reptarenavirus putative cap-binding domain and observed a high degree of similarity between the structures but also striking differences in the binding site composition and binding mode of the cap. In summary, we present evidence for a cap-binding site located within the L protein of a virus from the *Bunyavirales* order. This work also provides the basis for the design of specific compounds targeting RVFV cap-binding domain.

## Results

### Construct design and identification of a small domain in the RVFV L protein C-terminus

A secondary structure based sequence alignment of the C-termini of phlebo- and closely related banyangvirus L proteins was created to identify the region of the L protein, which might contain the potential cap-binding site (S1 Fig). Guided by the information about the structural composition of cap-binding domains from influenza virus PB2 and the putative cap-binding domain of California Academy of Sciences virus (CASV) L protein, we searched for several β-strands interspersed with one to three α-helices. The region between residues 1677 and 2008 of the RVFV L protein was identified as target region. We designed and cloned six different constructs from this area (putative cap-binding domain CBD 1–6) and tested for soluble expression in *Escherichia coli*. Construct details and expression data are summarized in S2 Fig. Two of the proteins could be solubly expressed (CBD2: residues 1677–1827; and CBD4: residues 1706–1827) but not well purified. Therefore, we designed further constructs (CBD 7–14) based on these soluble candidates and obtained two proteins suitable for crystallization experiments: CBD9 (residues 1719–1827) and CBD13 (residues 1706–1822).

### Crystallization and structure determination of a RVFV L protein C-terminal domain

**Fig 1:**
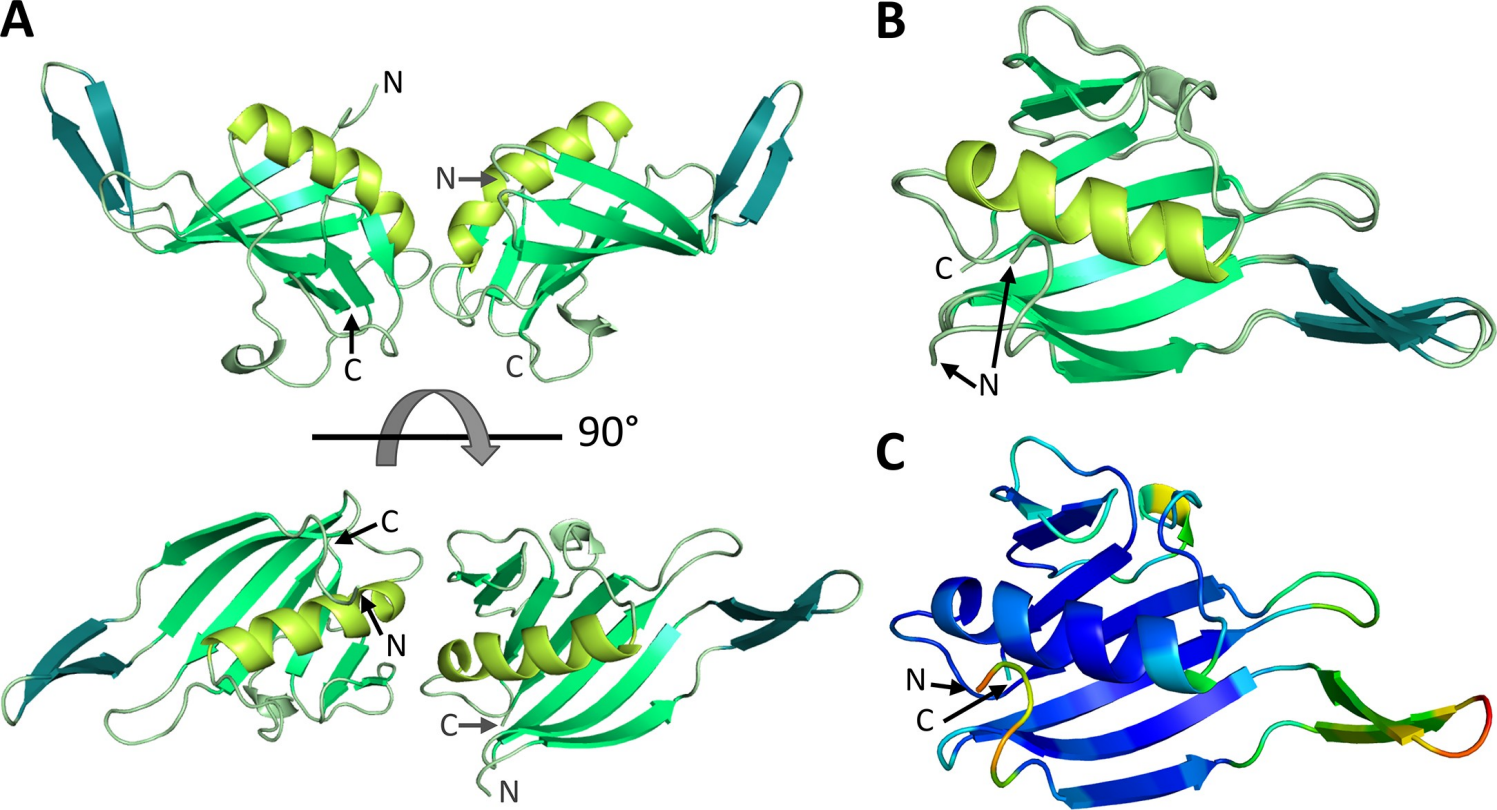
Atomic structure of RVFV L protein C-terminal domain. **A)** The structure of the protein dimer in the asymmetric unit is shown as a ribbon diagram in front and top view. Corresponding structural elements are shown in the same color (large β-sheet in medium green, long α-helix in lime green, β-hairpin in teal). N- and C-termini are labelled. **B)** Structures of RVFV CBD chain A and chain B are superimposed and depicted with the same color code. **C)** Representation of chain A of RVFV CBD as a ribbon diagram colored by B-factor with the highest observed B being 26 (orange) and the lowest 1 (dark blue).

Both CBD9 and CBD13 proteins were crystallized. During the purification process up to 6 mM of m^7^GTP was added, as it significantly reduced precipitation of the protein at low concentrations. Only CBD13 crystals were of good quality. This protein crystallized in space group P2_1_2_1_2_1_ with two molecules per asymmetric unit and the structure could be refined to a resolution of 1.5 Å (Fig 1A, S1 Table, PDB ID 6QHG). Thus, from now on CBD13 will be referred to as RVFV CBD. Except for the N-terminal residues 1706–1707 in chain B clear electron density was observed for the whole structure. RVFV CBD crystallized as a dimer (Fig 1A), which is not fully symmetric (Fig 1B), with a buried surface area of ~430 Å^2^ between the monomers (see also S3 Fig). The most prominent difference between the monomers is observed between residues 1718 and 1729, which constitute a β-hairpin. This β-hairpin has a slightly different position in both chains (Fig 1B, β-hairpin colored in teal). In solution, the protein appears to be monomeric, as revealed by size-exclusion chromatography (S4 Fig, buffer composition: 50 mM Na-phosphate, pH 6.5, 150 mM NaCl, 10% (w/v) glycerol). The structure of RVFV CBD consists of a β-sheet formed by seven β-strands, an additional β-hairpin as well as a long α-helix (Fig 1A and 1B). Overall the structure seems to be very rigid with low B-factors and only the β-hairpin shows some flexibility (Fig 1C).

### Identification and structural configuration of a cap-binding site in RVFV CBD

**Fig 2:**
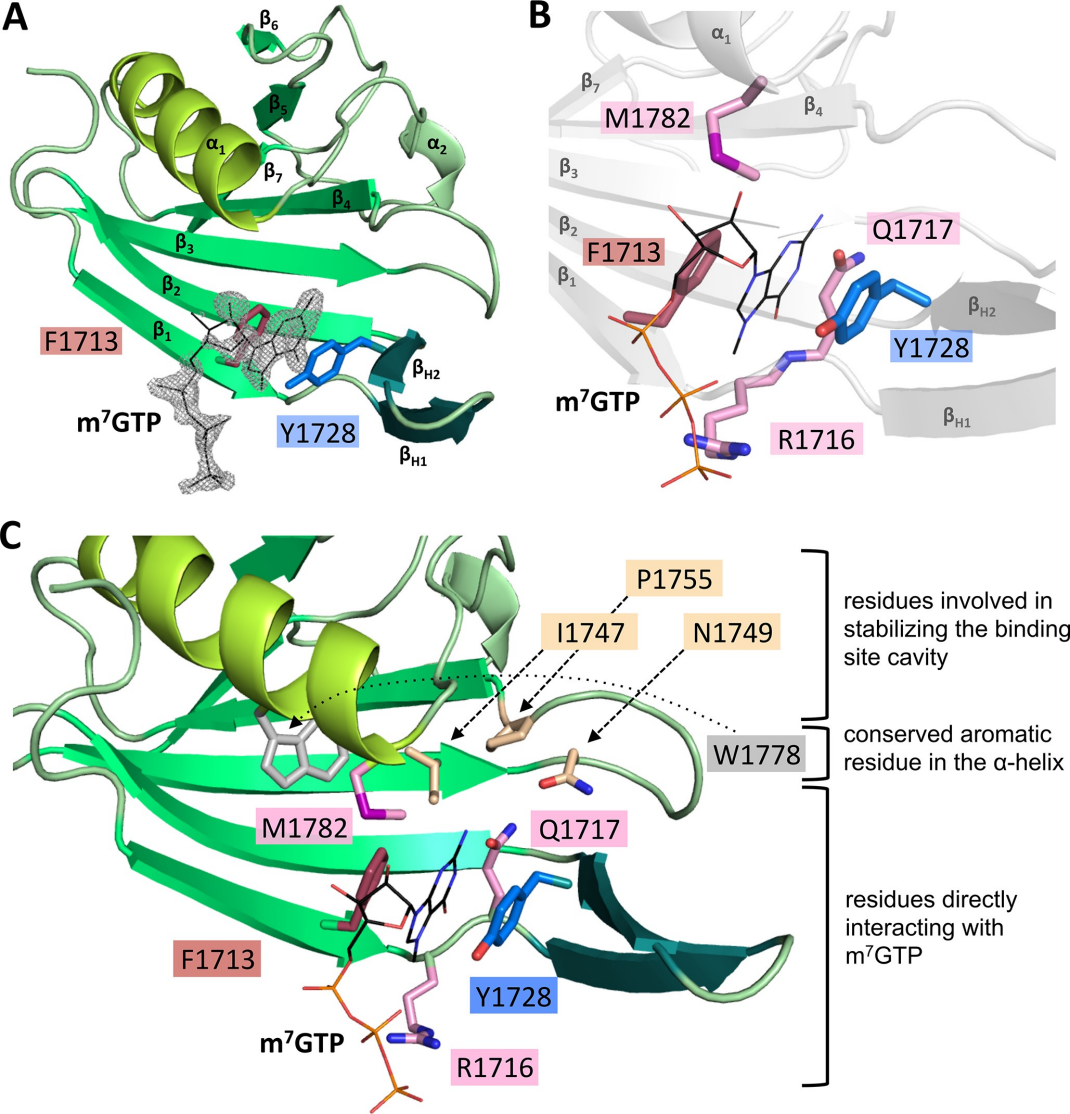
Interaction of m^7^GTP with RVFV CBD. **A)** The figure shows binding of m^7^GTP to RVFV CBD chain A in the crystal. RVFV CBD is presented as a ribbon diagram with the side chains of the two aromatic residues typical for binding of cap-structures shown as sticks. m^7^GTP is presented as lines and the surrounding electron density (2|Fo|-|Fc| omit map at 1.5σ) as grey mesh. Secondary structure elements are labelled. **B)** Amino acid side chains interacting with the m^7^GTP ligand (presented as lines) are shown as sticks. The structure of RVFV CBD chain A is indicated in grey. A detailed list of interactions is given in S2 Table. **C)** A close-up of the m^7^GTP binding site is presented. The protein is shown as ribbon diagram, involved side chains as sticks and the m^7^GTP as lines. The residues are grouped and colored according to A) and their role in the interaction with m^7^GTP.

During the purification optimization process, it was discovered that concentration of the protein to more than 4.5 mg/ml was only possible after addition of m^7^GTP, a cap-analogue. Thus, m^7^GTP was present in all crystallization setups and additional electron density corresponding to an m^7^GTP ligand was indeed clearly visible in both chains of the RVFV CBD structure (Fig 2A, S5A Fig). The m^7^GTP molecule was bound in a small, mainly hydrophobic pocket between two aromatic amino acid side chains, F1713 and Y1728 (Fig 2). Y1728 is located in the β-hairpin (β_H2_) and F1713 in the first β-strand (β_1_).

**Fig 3:**
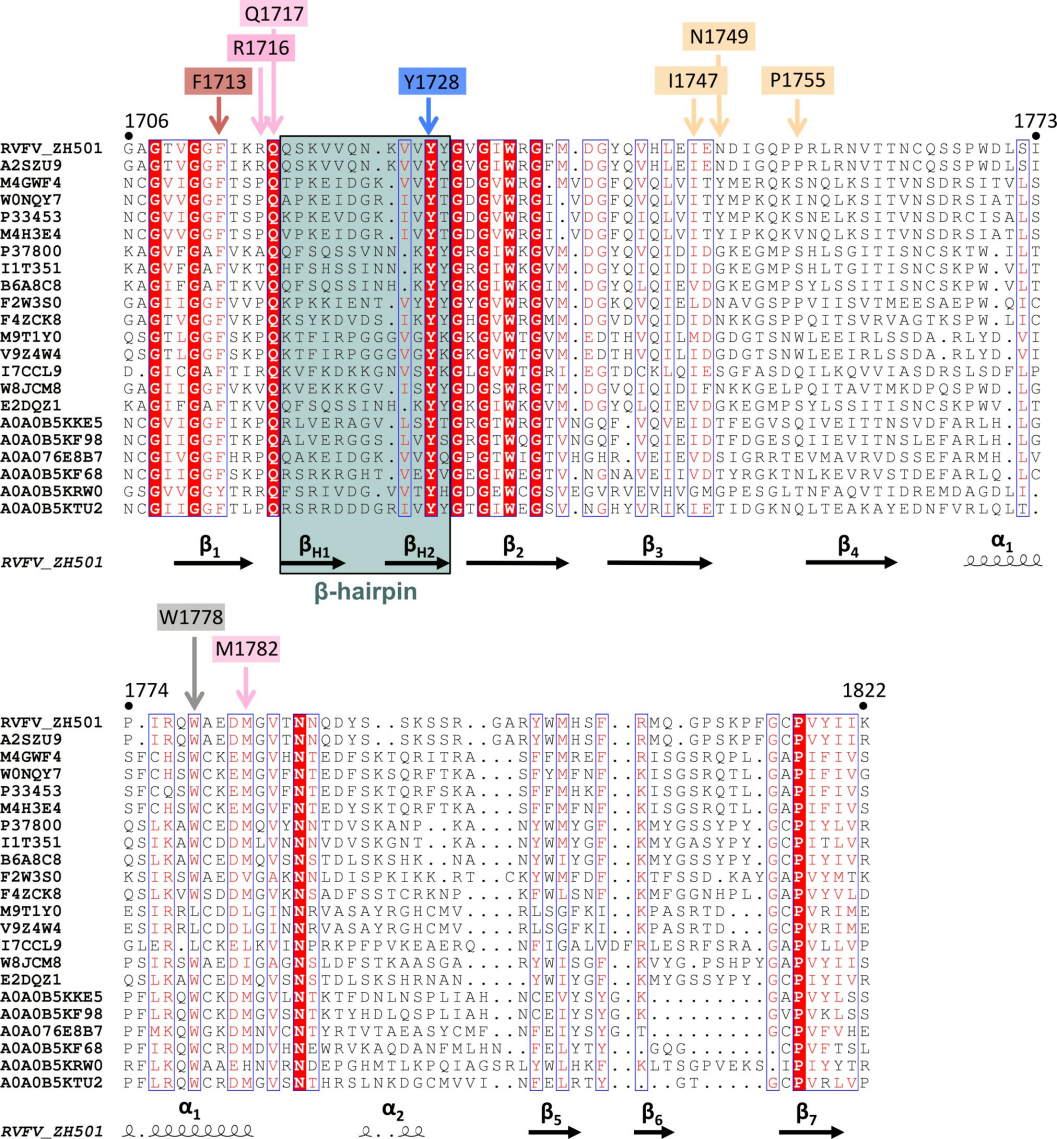
Alignment of the phlebo- and banyangvirus cap-binding domain. 22 sequences of phlebo- and banyangvirus cap-binding domains (Uniprot accession numbers are given) were aligned by manually combining ClustalOmega and PRALINE programs [53–56] and presented by ESPript (http://espript.ibcp.fr) [57]. Residue numbers refer to RVFV strain ZH-501 full-length L protein. Secondary structure elements of RVFV ZH-501 according to the RVFV CBD structure are depicted below the sequences. Residues involved in the cap-binding site are labelled with the same color code as used in Fig 2C.

The phosphate moiety of the cap structure interacts with R1716 side chain, which is extending from the loop between the first β-strand and the β-hairpin. Further contacts with the m^7^GTP involve M1782, interacting with the guanine moiety and ribose of the cap-structure, as well as Q1717, interacting with the guanine moiety (Fig 2B and 2C, S5B Fig, S2 Table). F1713, Y1728 and Q1717 are chemically conserved among phlebo- and banyangviruses, whereas for M1782 and R1716 the degree of conservation within the chemical class is about 95% and 20%, respectively (Fig 3). For chain A, some residues from neighboring molecules in the crystal also interact with the tri-phosphate of m^7^GTP. Due to the crystal symmetry, this is not the case for the m^7^GTP of chain B and the electron density for the ligand in chain B is less clear than in chain A (compare Fig 2A and S5A Fig). In summary, RVFV CBD was crystallized with an m^7^GTP ligand bound between two aromatic side chains. Such an arrangement is found in several cellular and viral cap-binding proteins, as reviewed by Fechter and Brownlee in 2005 [25].

### *In vitro* interaction studies of RVFV CBD with cap-structures

**Fig 4:**
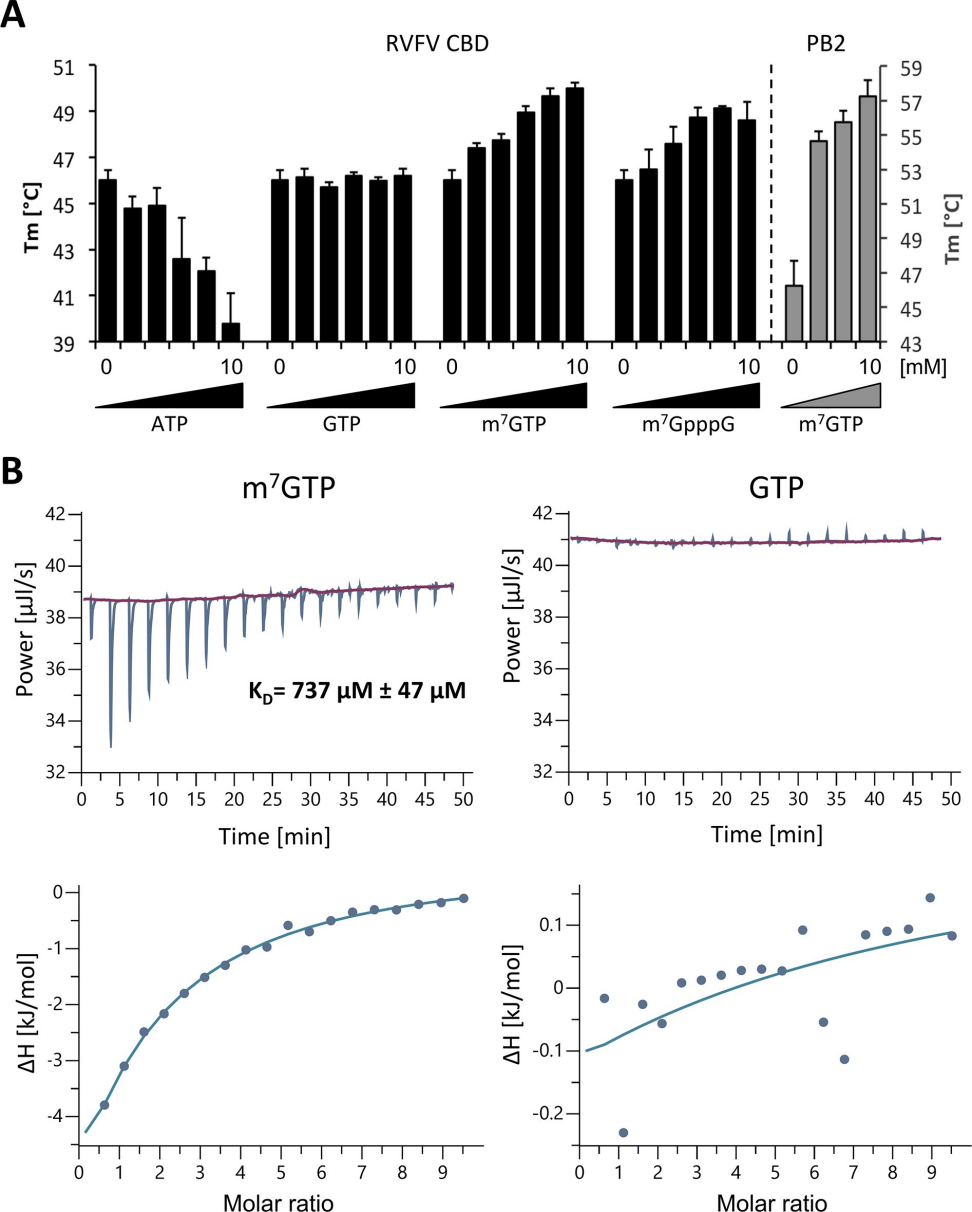
Examination of RVFV CBD cap-binding. **A)** Thermal stability of RVFV CBD and influenza A virus PB2 cap-binding domain was tested in presence and absence of different concentrations (1, 2, 5, 7.5, 10 mM) of m^7^GTP, m^7^GpppG, GTP and ATP. Melting temperatures are presented as mean and standard deviations of three independent measurements. Y-axis for RVFV CBD13 on the left (in black), y-axis for influenza A virus PB2 on the right (in grey). **B)** The affinity of RVFV CBD for m^7^GTP and GTP was measured by ITC at 25°C. RVFV CBD was in the cell at a concentration of 194 μM and m^7^GTP or GTP in the syringe at a concentration of 9.8 mM. The upper panel shows the raw data, the lower panel shows the integrated data fitted to a single-site binding model. The dissociation constant K_D_ is given as a mean and standard deviation of three independent measurements for m^7^GTP.

To verify that this binding pocket is specific for a methylated nucleotide, such as a cap-structure, we performed thermal stability assays using influenza virus PB2 cap-binding domain as a control. Addition of m^7^GTP to influenza virus PB2 cap-binding domain stabilized the protein, resulting in a higher melting temperature (ΔTm = 9°C in the presence of 10 mM m^7^GTP). We tested thermal stability of RVFV CBD in the presence of m^7^GTP, GTP, ATP and m^7^GpppG. We observed a specific stabilization of RVFV CBD by m^7^GTP as well as m^7^GpppG (ΔTm = 3–4°C) compared to no or even negative effects on protein stability after addition of GTP or ATP, respectively (Fig 4A). Isothermal titration calorimetry (ITC) measurements revealed a K_D_ of ~737 μM of RVFV CBD for m^7^GTP (Fig 4B), which is considerably higher than the K_D_ of 1.5 μM reported for m^7^GTP binding to influenza virus PB2 [26]. For GTP the K_D_ is too high to be determined by ITC under the same conditions as used for m^7^GTP (Fig 4B). Thus, ITC experiments confirmed specificity of RVFV CBD for m^7^GTP and not GTP. In summary, we could demonstrate a clear binding preference of RVFV CBD to m^7^GTP over GTP or ATP *in vitro*.

### Functional analysis of mutations of the cap-binding site in the context of the full-length L protein

**Fig 5:**
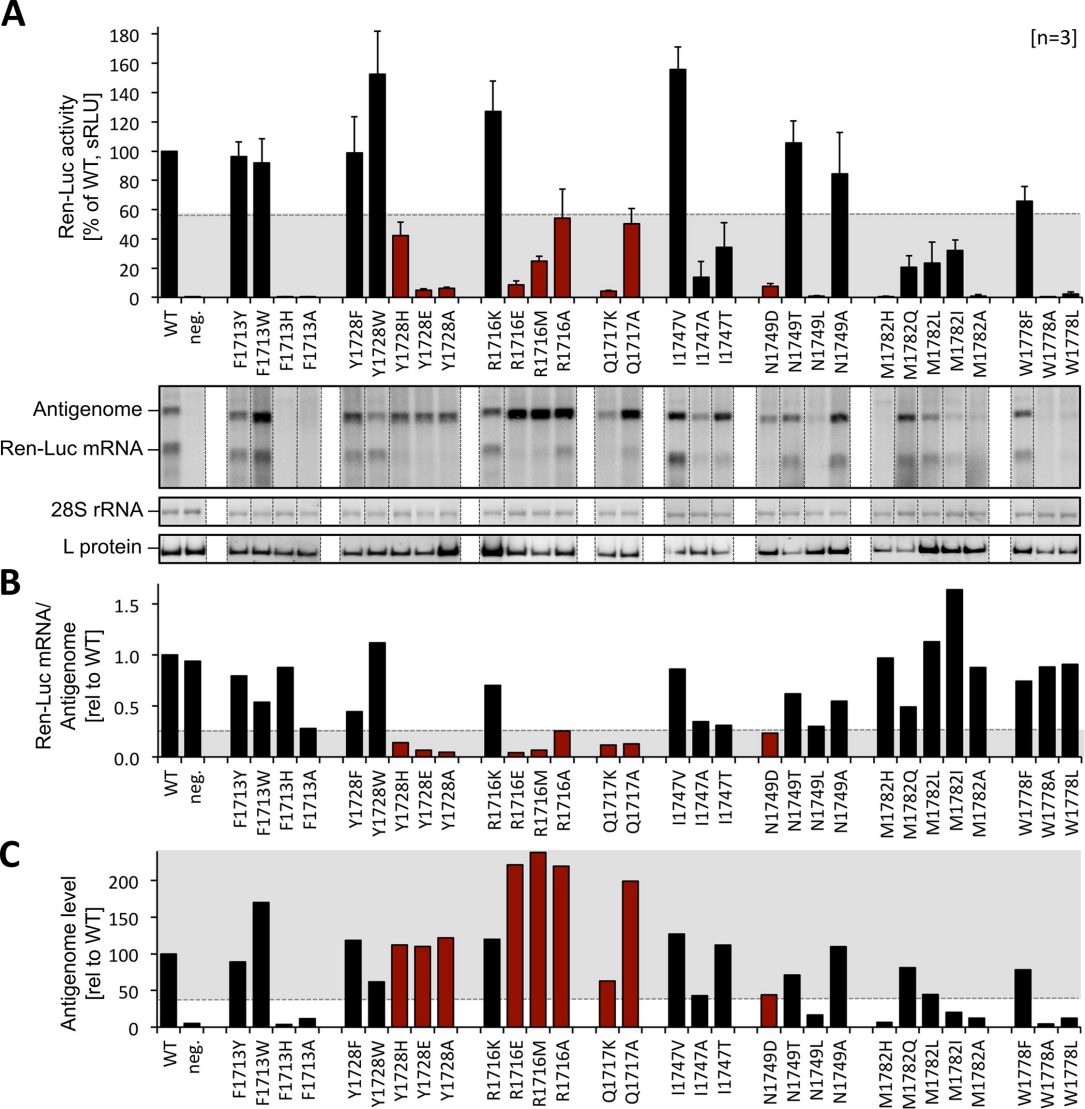
Minireplicon data for RVFV L protein mutants. **A)** Transcriptional activity of L protein mutants was measured via Ren-Luc reporter gene expression. The Ren-Luc activity, which has been normalized to the firefly activity (transfection control), is shown in the bar graph (mean and standard deviation of standardized relative light units [sRLU] as a percentage of the wild-type in 3 independent transfection experiments [n = 3]). Among other criteria, mutants with a selective defect in viral transcription are expected to show signals below 55% of wild-type activity (range is indicated by grey background). Synthesis of the antigenome and Ren-Luc mRNA was evaluated by northern blotting using a radiolabelled antisense riboprobe hybridizing to the Ren-Luc gene. A defective L protein with a mutation in the catalytic site of the RNA-dependent RNA polymerase served as a negative control (neg.). Signals on northern blots were quantified using ImageJ2 software [58]. All lanes displayed are from the same northern blot membrane (and same autoradiogram), dotted lines indicate cutting of the lanes for presentation reasons. The original blot is presented in S9 Fig. The data are also presented numerically in S3 Table. The methylene blue-stained 28S rRNA is shown as a marker for gel loading and RNA transfer. Immunoblot analysis of FLAG-tagged L protein mutants is also shown (L protein). Columns of mutants with a selective defect in viral transcription are colored in red. For experimental details see methods section. For quantitative analysis and definition of selective transcription defects see S3 Table. **B)** The column chart displays the signal ratio of mRNA to antigenome, reflecting viral transcription vs. replication, relative to the wild-type as calculated from the quantified northern blot signals. The expected range of signal ratio for mutants with a selective defect in mRNA production is indicated by grey background (≤ 0.25). Columns of mutants with a selective defect in viral transcription are colored in red. **C)** This column chart shows the antigenome levels determined by northern blot quantification relative to the wild-type level. L protein mutants with a selective transcription defect are expected to show wild-type like antigenome levels (≥ 40%, indicated by grey background). Columns of mutants with a selective defect in viral transcription are colored in red.

The RVFV minireplicon system [23] was used to test the effect of mutations in the cap-binding site on the ability of the L protein to transcribe and replicate the viral genome. By using an ambisense minigenome we were able to discriminate between (1) mRNA production, a process depending on the cap-snatching mechanism for priming, and (2) antigenome synthesis, which is independent of a primer. Synthesis of these two RNA species was measured via expression of renilla luciferase ((Ren-Luc) reflecting essentially viral transcription) and quantitative analysis of bands of antigenomic RNA and renilla luciferase mRNA on a northern blot. We know from previous experiments with the Lassa virus replicon system [22, 27, 28] that modification of L protein residues often affects the RNA polymerase function globally, which manifests as partial or complete defect in synthesis of all viral RNA species, i.e. replicative intermediates (antigenome) and mRNA. However, residues selectively important for viral transcription including cap-binding are expected to show a specific phenotype characterized by (i) reduced Ren-Luc signals indicating reduced mRNA synthesis, (ii) wild-type-like antigenome levels in the northern blot indicating functional RNA polymerase and (iii) a reduced mRNA-to-antigenome ratio as calculated from the quantitative northern blot data indicating a specific defect in transcription vs. replication (S3 Table). We exchanged eight residues of the cap-binding pocket and surrounding areas both for chemically similar and different amino acids (Fig 5, S3 Table). Aromatic residues F1713 and Y1728, relevant for stacking interaction with the m^7^GTP in the structure (Fig 2B and 2C), could be changed to other aromatic amino acids (F, Y, W) without loss in transcriptional activity, even though they are almost completely conserved among phlebo- and banyangviruses (Fig 3). Mutation of those aromatic residues to chemically different amino acids resulted either in a complete loss of L protein activity (F1713) or a selective defect in viral transcription (Y1728, Fig 5, columns of mutants with selective transcription defect are colored in red). Substitution of R1716 by chemically different amino acids also resulted in a selective transcription defect, which is somehow surprising considering the low degree of conservation among phlebo- and banyangviruses (Fig 3). A selective defect in viral transcription was also caused by mutations of residues Q1717 and N1749, although this effect was not observed for all substitutions. Residue M1782 could not be exchanged for other amino acids without a strong negative effect on general L protein function. We also included residue W1778 in our mutational study, because this is the only fairly conserved aromatic residue located in the α-helix, although it is not part of the cap-binding site (Fig 2C). W1778 is obviously of importance for general L protein function as mutations to non-aromatic amino acids resulted in a complete loss of L protein activity, whereas the exchange for a phenylalanine had no effect.

In summary, changes of the chemical properties of side chains directly interacting with m^7^GTP in the RVFV CBD structure mostly result in selective transcription defects in the context of the full-length L protein. These results support our structural data and underline the importance of the identified m^7^GTP-binding pocket for viral transcription.

### Overall structural comparison of RVFV CBD with related domains of other negative strand RNA viruses

**Fig 6:**
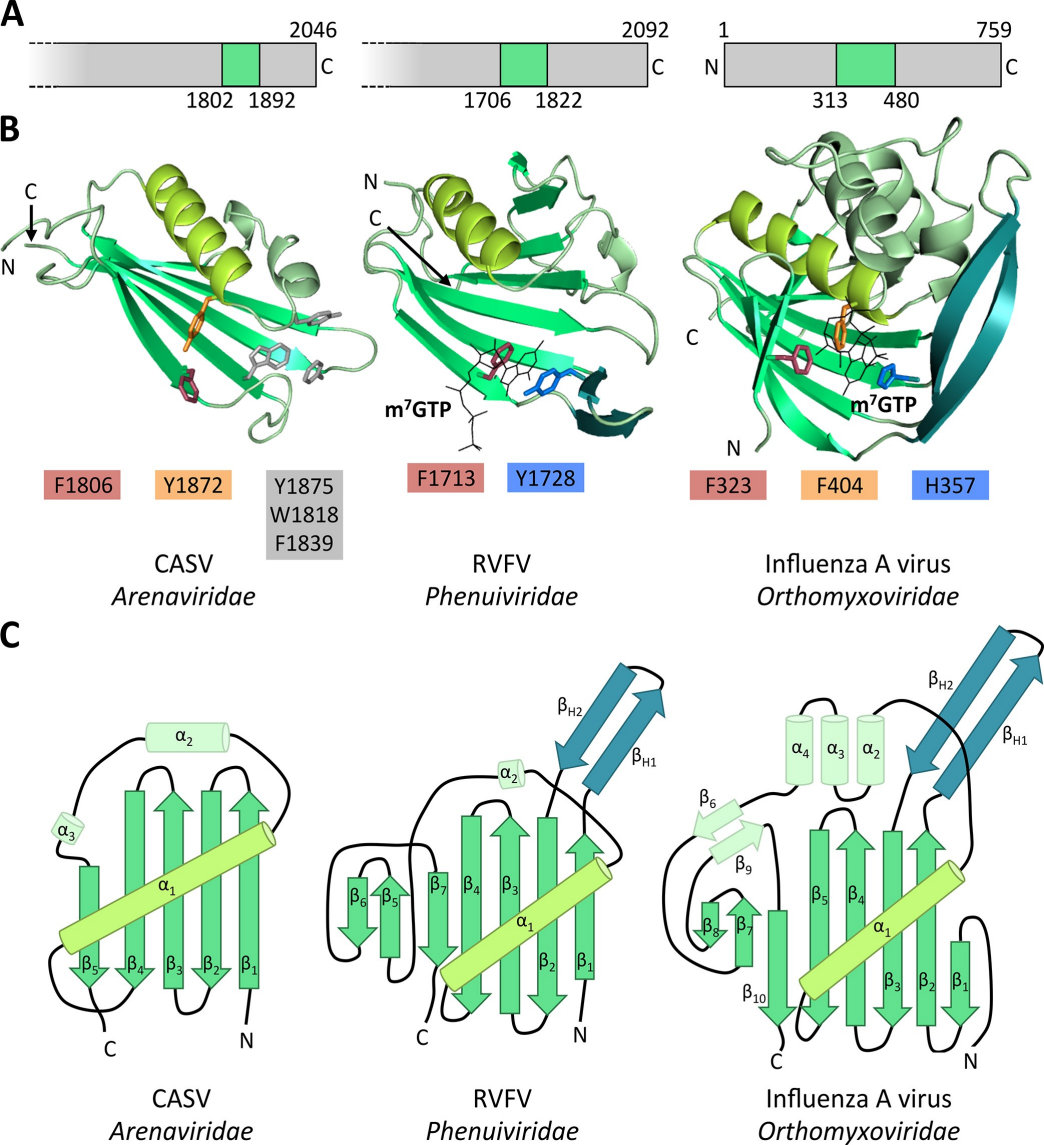
Comparison of RVFV CBD with influenza virus PB2 cap binding domain and CASV putative cap-binding domain. **A)** The location of the (putative) cap-binding domain in the respective L protein or PB2 protein is shown. Polymerase proteins are presented as grey bars, cap-binding domains are colored in green and the starting and ending residues of the domains are given as well as the length of the full-length L protein or PB2 protein, respectively. **B)** Structures of RVFV CBD chain A, influenza virus PB2 cap-binding domain (2VQZ) and CASV putative cap-binding domain (5MUZ) are shown as ribbon diagrams. Similar structural elements are depicted in the same color. Aromatic side chains of the (putative) cap-binding site are shown as sticks and corresponding side chains depicted in similar colors. See S8 Fig for a direct comparison of the (putative) cap-binding residues. **C)** Topology diagrams of the structures presented in B). Color coding also refers to B). Secondary structure elements are labelled.

The overall structure of RVFV CBD is similar to the known structures of influenza A virus PB2 cap-binding domain [29] and CASV putative cap-binding domain [21] (Fig 6B). Topology diagrams in Fig 6C provide an overview of the structural compositions of these proteins. They all consist of a large β-sheet packed against a long α-helix (α_1_). The β-hairpin present in the PB2 and RVFV CBD structures (β_H1+2_) is replaced by a long loop in the CASV structure. This β-hairpin is much longer in the PB2 structure compared to the RVFV structure. The large β-sheet is very twisted in PB2 and consists of 8 β-strands whereas it is quite straight and only composed of 5 β-strands in the CASV structure. RVFV CBD contains a moderately twisted 7-stranded β-sheet and is thus structurally an intermediate between CASV and PB2 structures. The PB2 cap-binding domain is larger than CASV putative cap-binding domain and RVFV CBD. The structural similarity between those three protein domains is striking, considering the low degree of sequence conservation (S6 and S7 Figs).

### Comparison of the RVFV cap-binding site residues with related proven or putative cap-binding sites

In many cap-binding proteins like eukaryotic initiation factor 4E (eIF4E), cellular cap-binding complex (CBC) or influenza virus PB2, the cap structures are bound between two aromatic side chains [25, 29]. This is also true for RVFV CBD. However, in the PB2 structure the two residues that bind the guanine moiety of the cap are located in the β-hairpin (H357 in β_H2_) and at the end of the long α-helix (F404 in α_1_), respectively (Fig 6B, right panel). Additionally, the ribose interacts with a phenylalanine side chain (F323 in β_1_) extending from the first β-strand of the large β-sheet (Fig 6B, right panel, S5C Fig). In RVFV CBD, no aromatic amino acid is present at the end of the long α-helix (α_1_). Instead, the guanine moiety is bound between two aromatic side chains extending from the β-hairpin (Y1728 in β_H2_) and the first β-strand (F1713 in β_1_) (Fig 6B, middle panel, Fig 2C, S5B Fig), which are also highly conserved among phlebo- and banyangviruses (Fig 3). The latter residue, F1713, corresponds to F323 of influenza virus PB2, which in PB2 interacts with the ribose. In RVFV CBD methionine 1782 at the end of the long α-helix (α_1_) takes the role of positioning the ribose (Fig 2C, S8 Fig). In case of the CASV C-terminal domain, there is a conserved aromatic residue (Y1872 in α_1_) located at the end of the long α-helix, similar to F404 in influenza virus PB2, but no second residue in a conformation and distance suitable for a stacking interaction with a cap-structure is present (Fig 6B, left panel). Among phlebo- and banyangviruses, we found only one aromatic residue near the end of the long α-helix (α_1_), W1818, which is conserved in about 85% of the analyzed sequences (Fig 3). The tryptophan side chain, however, would be unable to interact with m^7^GTP in our structure as it is covered by M1782 (Fig 2C).

In summary, although the tertiary structure of the (putative) cap-binding domains of influenza virus, CASV, and RVFV is highly conserved, the atomic details of the cap binding sites differ significantly.

## Discussion

Cap-snatching was first discovered in influenza virus [30] and is an attractive drug target, because the process is essential for virus amplification and the enzymes involved are virus encoded. This mechanism comprises two targets: the endonuclease and the cap-binding site. However, whereas several structures of bunyavirus endonucleases have been solved and the enzyme has been characterized relatively well [7, 16, 18–21, 31], structural data on the cap-binding site of bunyaviruses is rare and functional data is missing. The publication of the structure of a reptarenavirus L protein domain structurally similar to influenza virus PB2, but deficient of cap-binding activity raised the question whether the observed inability of cap-binding truly reflects the situation in mammarenaviruses and possibly all viruses of the *Bunyavirales* order [21].

Here we present the structure of a small globular domain located in the C-terminal region of RVFV L protein along with the first evidence for a functional cap-binding site within the bunyavirus L protein. The new structure is similar to influenza virus PB2 cap-binding domain and CASV putative cap-binding domain. The RVFV protein specifically binds to a cap-analogue *in vitro* and upon co-crystallization the cap-analogue could be detected bound between two aromatic side chains, as it is typical for several cellular and viral cap-binding proteins [25]. Still, the residues involved in the interactions with the ligand differ significantly between influenza virus PB2 and RVFV cap-binding domain. These findings pose the following questions: (1) Is a functional cap-binding domain a common feature of all viruses within the B*unyavirales* order? (2) Will it be possible to design broad-spectrum inhibitors targeting the cap-binding domain of segmented negative sense RNA viruses despite the observed differences in the binding mode of m^7^GTP?

The presented data support the hypothesis that bunyavirus L proteins are functionally and structurally equivalent to the concatenation of influenza virus polymerase subunits in the order PA-PB1-PB2 [17]. The presence of a β-hairpin in the RVFV structure, similar to PB2 but different from the CASV structure, suggests that the new structure might be more closely related to influenza virus cap-binding domain from an evolutionary perspective, which is contrary to what is described in the literature [32], although it is worth mentioning that most of the phylogenetic analyses for the L gene are based on the conserved RNA-dependent RNA polymerase region. The evolutionary relation of the cap-binding domains will be difficult to prove, as the observed similarity is not obvious from the sequence level (S6 and S7 Figs).

Our data illustrate the structural flexibility of proteins with the same function, even though they are evolutionarily closely related. This is probably the reason why it is unlikely to reliably predict the cap-binding site in L proteins of different families within the *Bunyavirales* order from the sequence level. The C-terminal part of the L protein of *Bunyavirales* is poorly conserved, making sequence alignments challenging. Furthermore, the location of the proven or putative cap-binding domain within the polymerase proteins differs between RVFV, CASV and influenza virus, which makes a correct alignment of these sequences even more difficult. Whereas for CASV the putative cap-binding domain is between 250 and 150 amino acids distance from the C-terminus, for RVFV this domain is between 390 and 270 residues away from the C-terminus (Fig 6A). The evolutionary context of this observation will be interesting to investigate in the future. We created an alignment of the proven and putative cap-binding domains of phlebo- and closely related banyangviruses, as well as arenaviruses and influenza A virus based on the observed structural similarities (S6 Fig). This alignment has to be interpreted with caution, as it demonstrates the very low degree of sequence conservation in this domain (see identity/ similarity matrix in S7 Fig). The lack of a reasonable alignment makes it highly unlikely to model the cap-binding domains of other bunyaviruses based on the known structures. This underlines the need to localize and solve structures of further putative cap-binding domains in order to fully understand the cap-snatching mechanism of bunyaviruses.

For the development of potentially broad-spectrum inhibitors against viruses from the *Bunyavirales* order as well as influenza viruses, the structures of the cap-binding cavities are essential. The binding site topology of the RVFV L protein cap-binding domain, however, is different from the PB2 cap-binding site: The corresponding essential aromatic residue from the end of the long α-helix (α_1_) in PB2 (F404) is absent in the RVFV structure. Instead, this function is taken over by an aromatic residue (F1713) located in the first β-strand (β_1_) of the sheet. The equivalent residue in influenza virus PB2 (F323) is responsible for stacking the ribose moiety of the cap (S8 Fig). Interestingly, the aromatic residue at the end of the long α-helix (α_1_) is present in the CASV domain (Y1872), although no reasonable candidate as a second partner for an aromatic sandwich is apparent (Fig 6B, S8 Fig).

We were able to demonstrate binding of the RVFV CBD to m^7^GTP, a cap-analogue. In a thermal stability assay we observed stabilization of RVFV CBD by m^7^GTP. Additionally, we determined the K_D_ of the low affinity interaction between m^7^GTP and RVFV CBD, which was considerably high with 737 μM. For influenza A virus PB2, both the shift in melting temperature in presence of m^7^GTP (ΔT_m_ <9°C, Fig 4) and the K_D_ for interaction with m^7^GTP, which was shown to be ~1.5 μM *in vitro* [26], point to a higher affinity interaction with the m^7^GTP compared to RVFV cap-binding domain. This difference is not surprising, considering the few interactions observed between the RVFV L protein fragment and the ligand in the structure (compare S5B and S5C Fig, S2 Table) and the fact that we are working with an isolated L protein domain. The affinity of RVFV full-length L protein for capped mRNAs has to be much higher to compete for the ligand with other cap-binding proteins inside the cytoplasm. The K_D_ for the interaction of human eIF4E with m^7^GTP is ~0.87 μM [33]. This suggests that there have to be more residues in RVFV L protein interacting with the cap-structure or the first nucleotides of the capped mRNA apart from the small domain presented here. This is conceivable as for influenza virus PB2 residues of the cap-binding adjacent, so-called mid-link domain have been shown to interact with the capped RNA [34]. Alternatively, other mechanisms may support RVFV cap-binding activity. As an example, arenavirus Z protein has been shown to interact with eIF4E and to down-regulate its affinity for cap-structures [35]. Although most bunyaviruses do not contain a Z gene, similar scenarios involving viral proteins are conceivable. Furthermore, bunyavirus non-structural proteins have been shown to regulate host transcription and replication [36]. Additionally, cellular interaction partners of the L protein might play a role in the cap-snatching process as well as the cellular localization of viral transcription processes.

For influenza virus several compounds targeting the two functions involved in cap-snatching, the endonuclease and cap-binding site, have been reported over the past years [37–41], two of those having succeeded in clinical trials [42, 43]. An inhibitor of influenza endonuclease has been shown to be also effective against the homologous enzyme of La Crosse bunyavirus *in vitro* [44], which demonstrates the possibility to develop broad-spectrum inhibitors against the cap-cleaving endonucleases of influenza viruses and bunyaviruses. However, the observed differences in the topology of the cap-binding sites of influenza virus and RVFV are likely to be a challenge for the design of broad-spectrum inhibitors targeting cap-binding. Further structures of *Bunyavirales* cap-binding domains are essential to evaluate this option. The structural and functional data presented here, provide evidence for the existence of a functional cap-binding domain in bunyavirus L protein essential for viral transcription as well as the foundation for structure based drug development against RVFV cap-snatching mechanism.

## Materials and methods

### Cloning, expression and purification of RVFV L protein C-terminus

Based on an alignment of phlebo- and banyangvirus L protein C-terminal sequences, we designed constructs for RVFV L protein (strain ZH-501, Uniprot accession: A2SZS6) covering the area between residues 1677 and 2008. All sequences were cloned into pOPINF vectors [45] using the NEBuilder HiFi DNA Assembly Cloning Kit (New England BioLabs). Proteins were expressed in *E*. *coli* strain BL21 Gold (DE3) (Novagen) at 17°C overnight using TB medium and 0.5 mM isopropyl-β-D-thiogalactopyranosid for induction. After pelleting, the cells were resuspended in 50 mM Na-phosphate pH 7.5, 100 mM NaCl, 10 mM imidazole, Complete protease inhibitor EDTA-free (Roche), 0.4% (v/v) triton X-100 and 0.025% (w/v) lysozyme and subsequently disrupted by sonication. The protein was purified from the soluble fraction after centrifugation by Ni affinity chromatography. Buffers containing either 50 mM imidazole and 1 M NaCl or 50 mM imidazole and 100 mM NaCl were used for the washing steps and another buffer with 500 mM imidazole for the elution of the protein. Eluted protein was immediately diluted with 20 mM Na-phosphate pH 6.5 followed by removal of the N-terminal His-tag by a GST-tagged 3C protease at 4°C overnight. The protein was further purified by passing through an anion exchange chromatography column (HiTrap Q FF, GE Healthcare) and subsequent cation exchange chromatography (Hi Trap SP FF, GE Healthcare, loading buffer: 50 mM Na-phosphate pH 6.5, 50 mM NaCl, 10% (w/v) glycerol, elution with salt gradient up to 1M NaCl). After addition of up to 3 mM m^7^GTP the protein was concentrated for a final size exclusion chromatography (Superdex 200, 50 mM Na-phosphate, pH 6.5, 150 mM NaCl, 10% (w/v) glycerol). Purified proteins were concentrated using centrifugal devices with addition of up to 6 mM m^7^GTP (final concentration), flash frozen in liquid nitrogen, and stored in aliquots at –80°C. For thermal stability assays and isothermal titration calorimetry purification procedure was done without addition of m^7^GTP resulting in lower protein concentrations of max. 4.5 mg/ml.

### Production of seleno-methionine labelled protein

Protein expression was done in M9 minimal medium [46] supplemented with 1 mM MgSO_4_, 0.4% glucose, 0.0005% thiamine and 200 μM FeSO_4_ at 17°C overnight. Incorporation of seleno-methionine was achieved by metabolic inhibition of methionine biosynthesis in *E*. *coli* prior to addition of seleno-methionine and induction with 1 mM isopropyl-β-D-thiogalactopyranosid. Cells were harvested and the labelled protein was purified as described but in presence of 5 mM β-mercaptoethanol for Ni affinity purification and 10 mM DTT for the remaining purification steps.

### Crystallization and structure determination

RVFV CBD13 protein was produced with seleno-methionine labelling, CBD9 only as native protein. Protein crystals of CBD9 grew at 8 mg/ml protein concentration in 21% PEG 4000, 10% glycerol, 12% isopropanol and 100 mM Na-Citrate pH 6.0 in a sitting drop vapor diffusion setup at 20°C. Protein crystals of CBD13 grew at 9 mg/ml protein concentration in 24% PEG 2000 MME, 200 mM Trimethylamine N-oxide, 2 mM TCEP, 5 mM dithiothreitol, 2 mM m^7^GTP and 100 mM Tris, pH 8.5 in a sitting drop vapor diffusion setup at 20°C. Crystals were flash frozen in liquid nitrogen without cryo protectants in case of CBD13 and with 25% ethylenglycol in reservoir solution for CBD9. Datasets were obtained at beamline P13 of PETRA III at Deutsches Elektronen Synchrotron (DESY), Hamburg, Germany. Datasets were processed with iMosflm [47] and the RVFV CBD13 structure was solved by the single anomalous dispersion method using PHENIX AutoSol [48]. The structure was refined by iterative cycles of manual model building in Coot [49] and computational optimization with PHENIX [48]. Visualization of structural data was done using the PyMOL Molecular Graphics System, Version 1.7 Schrödinger, LLC.

### Thermal stability assay

Thermal stability of RVFV CBD13 was measured by thermofluor assay [50]. The assay contained a final concentration of 15 μM of CBD13 protein, 20 mM Na-phosphate pH 6.5, 100 mM NaCl, SYPRO-Orange (final dilution 1:1000) and either no additive or between 1 and 10 mM of m^7^GTP, m^7^GpppG, GTP or ATP. Thermal stability of influenza A virus PB2 cap-binding domain was assessed at a final protein concentration of ~10 μM in the same setup.

### Isothermal titration calorimetry

Affinity of RVFV CBD13 to m^7^GTP and GTP was measured by isothermal titration calorimetry (ITC) using a MicroCal PEAQ-ITC instrument (Malvern Panalytical). The instrument was calibrated using a control reaction of Ca^2+^ binding to EDTA as supplied by Malvern Pananalytical. Proteins were dialyzed overnight at 4°C against 50 mM Na-phosphate, pH 6.5, 150 mM NaCl, 10% (w/v) glycerol. Ligand m^7^GTP was dissolved and GTP was diluted in the exact same dialysis buffer. Titrations were done with slightly different setups: (1) 128 μM CBD13 in the cell and 3.8 mM m^7^GTP in the syringe at 20°C with 13 injections of 3 μl (first injection 0.5 μl), (2) 194 μM CBD13 in the cell and 9.8 mM m^7^GTP in the syringe at 25°C with 19 injections of 2 μl (first injection 0.5 μl), or (3) 194 μM CBD13 in the cell and 9.8 mM GTP in the syringe at 25°C with 19 injections of 2 μl (first injection 0.5 μl). Spacing between injections was constant with 150 seconds for all measurements. Data were analyzed and fitted with the respective PEAQ ITC evaluation software (Malvern) applying a single site binding model and fixing the stoichiometry value to 1.

### Testing of L protein mutants in the RVFV minireplicon system

The experiments were performed in the context of the T7 RNA polymerase-based RVFV ambisense minireplicon system essentially as described by Jérôme *et al*. [23]. L gene mutants were generated by mutagenic PCR using pCITE-L as a template. The PCR products containing the functional cassette for expression of mutant L protein were purified, quantified spectrophotometrically, and used for transfection without prior cloning. The presence of the artificial mutation was ascertained by sequencing. BSR-T7/5 cells stably expressing T7 RNA polymerase [51] (kindly provided by Ursula Buchholz and Karl-Klaus Conzelmann) were transfected per well of a 24-well plate with 250 ng of L gene PCR product, 750 ng of RVFV minigenome plasmid expressing Renilla luciferase (Ren-Luc), 500 ng of pCITE-NP expressing NP, and 10 ng of pCITE-FF-Luc expressing firefly luciferase as an internal transfection control. One day after transfection, total RNA was purified using an RNeasy Mini Kit (Qiagen) for northern blotting or cells were lysed in 100 μl of passive lysis buffer (Promega) per well, and assayed for firefly luciferase and Ren-Luc activity using the dual-luciferase reporter assay system (Promega). Ren-Luc levels were corrected with the firefly luciferase levels (resulting in standardized relative light units [sRLU]) to compensate for differences in transfection efficiency or cell density.

For northern blot analysis, 500 ng of RNA was separated in a 1.5%-agarose–formaldehyde gel and transferred onto a Roti-Nylon plus membrane (Roth). Blots were hybridized with a ^32^P-labelled antisense riboprobe targeting the Ren-Luc gene, and RNA bands were visualized by autoradiography using a Typhoon scanner (GE Healthcare).

To verify expression of L protein mutants, BSR-T7/5 cells in a well of a 24-well were transfected with 500 ng of PCR product expressing L protein mutants tagged at the C-terminus with a 3xFLAG sequence. To enhance the expression level, the cells were additionally inoculated with Modified Vaccinia virus Ankara expressing T7 RNA polymerase (MVA-T7) [52]. Cytoplasmic lysate was separated in a 3–8% Tris-acetate polyacrylamide gel, transferred to nitrocellulose membrane (Whatman), and detected by immunoblotting using peroxidase-conjugated anti-FLAG M2 antibody (1:10,000) (A8592; Sigma-Aldrich). L protein bands were visualized by chemiluminescence using SuperSignal West Femto substrate (Pierce) and a FUSION SL image acquisition system (Vilber Lourmat).

## Supporting information

S1 Table Crystallographic data and refinement statistics. (PDF)

S2 Table List of interactions of RVFV CBD chain A with m7GTP generated by PDBsum. The table lists interactions between atoms in form of hydrogen bonds as well as non-bonded contacts between RVFV CBD chain A (representing also chain B) and co-crystallized m^7^GTP. The list was generated using PDBsum [59].
(PDF)

S3 Table Analysis of L protein mutants using the RVFV minireplicon system. (PDF)

S1 Fig Alignment of phlebo- and banyangvirus L protein C-terminal sequences. The figure presents the secondary-structure based alignment of the L protein C-terminal sequences of 22 phlebo- and banyangviruses (Uniprot accession numbers are given). Chemically similar residues are depicted in the same color. The corresponding secondary structure prediction was calculated by Jpred4 [60] and is depicted below the sequences (β-sheets in red, α-helices in blue, asterisks for loops). N- and C-terminal boundaries of tested constructs are indicated by arrows above the sequences. N- and C-termini of RVFV CBD13 are indicated with red arrows. All numbers refer to RVFV strain ZH-501 full-length L protein.
(TIF)

S2 Fig Overview of constructs tested for RVFV L protein C-terminus. An overview of all RVFV constructs designed and tested is given: the respective N- and C-termini, rating of protein solubility from insoluble (-) and somehow soluble (+) up to highly soluble (++), as well as information about suitability for crystallization (no, yes, n.d. = not determined).
(TIF)

S3 Fig Dimer interface in the crystal structure. The figure was taken from PDBsum [59] to summarize the interactions observed between the two monomers in the crystal structure.
(TIF)

S4 Fig Size-exclusion chromatography for RVFV CBD. An example of a size-exclusion chromatogram is given with the absorption at wavelength 280 nm being monitored. Chromatography was performed on a Superdex 200 16/60 HiLoad column at 4°C with a buffer containing 50 mM Na-phosphate, 100 mM NaCl and 10% glycerol. Elution volumes of calibration proteins are indicated by dotted lines and labelled with the respective molecular weight of the protein. The estimated molecular weight of the protein in the peak fraction was calculated based on the column calibration and is displayed below the graph.
(TIF)

S5 Fig Electron density map for m7GTP in RVFV CBD chain B and ligand plots. **A)** The figure shows binding of m^7^GTP to CBD13 chain B in the crystal. CBD13 is presented as a ribbon diagram with the side chains of the two aromatic residues typical for binding of cap-structures shown as sticks. m^7^GTP is presented as lines and the surrounding electron density (2|Fo|-|Fc| omit map at 1.5σ) as grey mesh. Secondary structure elements are labelled. **B)** A ligand plot for interaction of m^7^GTP with RVFV CBD chain A is presented. The plot was generated by PDBsum [59] and modified to also include M1782. A detailed list of interactions is displayed in S2 Table **C)** A ligand plot for interaction of m^7^GTP with influenza virus PB2 (PDB:2VQZ, chain A) is presented. The plot was generated by PDBsum [59] and modified for presentation reasons.
(TIF)

S6 Fig Alignment of the (putative) cap-binding domains of phlebo- and banyangviruses, arenaviruses and influenza A virus. This figure presents an alignment of the (putative) cap-binding domains of 2 reptarenaviruses (light green label), 4 mammarenaviruses (dark green label), 7 phleboviruses and 1 banyangvirus (blue label) and influenza A virus (red label). The alignment was essentially based on predicted secondary structures and calculated using PRALINE software [53, 56] but required major manual adjustments. Graphical presentation of the alignment was done using ESPript (http://espript.ibcp.fr) [57]. The secondary structure of RVFV CBD (PDB 6QHG, on blue background) and influenza virus cap-binding domain (PDB 2VQZ, on red background) are displayed above and below the sequences, respectively. Secondary structure elements are labelled according to Fig 6. Numbering refers to RVFV strain ZH-501 full-length L protein.
(TIF)

S7 Fig Identity and similarity matrix corresponding to alignment from S6 Fig. Identity and similarity matrices calculated by SIAS online tool (http://imed.med.ucm.es/Tools/sias.html) are presented for selected sequences of the alignment displayed in S6 Fig.
(TIF)

S8 Fig Comparison of the (putative) cap-binding residues. The amino acids (putatively) interacting with an m^7^GTP ligand are compared between the structures of CASV L-Cterm (5MUZ), RVFV CBD (6QHG) and influenza virus PB2 (2VQZ) according to Fig 6. The location of the residues is given and structurally corresponding residues are marked with the same color. As for the CASV putative cap-binding domain no m^7^GTP binding has been demonstrated, the displayed residues solely correspond to structural homologs.
(TIF)

S9 Fig Original northern blot membrane. This figure provides the original northern blot autoradiogram presented in Fig 5.
(TIF)
