# Supplementary material for: Structure of a functional cap-binding domain in Rift Valley fever virus L protein
Source: PLoS Pathog. 2019 May 28;15(5):e1007829. doi: 10.1371/journal.ppat.1007829 (PMC6555543; doi:10.1371/journal.ppat.1007829)
Supplement: S1 Table — (PDF) [file ppat.1007829.s001.pdf]

S1 Table. Crystallographic data and refinement statistics.

	**RVFV CBD_{SeMet}**
**Data collection**	
Synchrotron beamline	PETRA P13
Wavelength (Å)	0.979
Resolution (Å)	67.99 - 1.48 (1.53 - 1.48)
Space group	$P2_12_12_1$
Cell dimensions	
a, b, c (Å)	31.5798, 53.6807, 135.987
α, β, γ (°)	90, 90, 90
Total reflections	468412 (23847)
Unique reflections	39006 (3785)
Multiplicity	12.0 (12.6)
Completeness (%)	0.99 (0.98)
Mean I/sigma(I)	10.0 (4.2)
Wilson B-factor	3.93
R-merge	0.113 (0.403)
R-meas	0.123 (0.437)
**Refinement**	
Resolution (Å)	42.13-1.48
Reflections used in refinement	38898 (3785)
Reflections used for R-free	1888 (162)
R-work	0.1519 (0.1507)
R-free	0.1936 (0.2159)
No. of atoms	
protein	1908
ligand/ ion	83
Water molecules	236
Average B-factors (Å^2)	
protein	9.50
ligand	23.30
solvent	26.31
R.m.s deviations	
bond lengths (Å)	0.018
bond angles (°)	0.90
Ramachandran (%)	
favored	98
allowed	1.7
outliers	0
Rotamer outliers (%)	0.97
**PDB code**	**6QHG**
