# Supplementary material for: Structure of a functional cap-binding domain in Rift Valley fever virus L protein
Source: PLoS Pathog. 2019 May 28;15(5):e1007829. doi: 10.1371/journal.ppat.1007829 (PMC6555543; doi:10.1371/journal.ppat.1007829)
Supplement: S2 Table — The table lists interactions between atoms in form of hydrogen bonds as well as non-bonded contacts between RVFV CBD chain A (representing also chain B) and co-crystallized m7GTP. The list was generated using PDBsum [59]. (PDF) [file ppat.1007829.s002.pdf]

S2 Table. List of interactions of RVFV CBD chain A with m^7GTP generated by PDBsum.

Hydrogen bonds

	<----	ATOM 1			----->		<---	ATOM 2			----->	
	Atom no.	Atom name	Res name	Res no.	Chain		Atom no.	Atom name	Res name	Res no.	Chain	Distance
1	84	NH1	ARG	1716	A	-->	969	O2B	MGT	2001	A	2.71
2	85	NH2	ARG	1716	A	-->	965	O3G	MGT	2001	A	2.98
3	86	N	GLN	1717	A	-->	989	O6	MGT	2001	A	2.75
4	93	OE1	GLN	1717	A	<--	990	N1	MGT	2001	A	2.67
5	93	OE1	GLN	1717	A	<--	992	N2	MGT	2001	A	2.95

Non-bonded contacts

	<----	ATOM 1			----->		<---	ATOM 2			----->	
	Atom no.	Atom name	Res name	Res no.	Chain		Atom no.	Atom name	Res name	Res no.	Chain	Distance
1	54	CD2	PHE	1713	A	---	985	N7	MGT	2001	A	3.83
2	54	CD2	PHE	1713	A	---	987	C5	MGT	2001	A	3.78
3	56	CE2	PHE	1713	A	---	987	C5	MGT	2001	A	3.66
4	56	CE2	PHE	1713	A	---	988	C6	MGT	2001	A	3.85
5	56	CE2	PHE	1713	A	---	994	C4	MGT	2001	A	3.8
6	57	CZ	PHE	1713	A	---	988	C6	MGT	2001	A	3.89
7	57	CZ	PHE	1713	A	---	990	N1	MGT	2001	A	3.81
8	76	CA	ARG	1716	A	---	989	O6	MGT	2001	A	3.67
9	77	C	ARG	1716	A	---	989	O6	MGT	2001	A	3.67
10	79	CB	ARG	1716	A	---	986	CM7	MGT	2001	A	3.8
11	80	CG	ARG	1716	A	---	986	CM7	MGT	2001	A	3.76
12	83	CZ	ARG	1716	A	---	965	O3G	MGT	2001	A	3.62
13	83	CZ	ARG	1716	A	---	969	O2B	MGT	2001	A	3.66
14	83	CZ	ARG	1716	A	---	986	CM7	MGT	2001	A	3.81
15	84	NH1	ARG	1716	A	---	965	O3G	MGT	2001	A	3.54
16	84	NH1	ARG	1716	A	---	967	PB	MGT	2001	A	3.76
17	84	NH1	ARG	1716	A	---	968	O1B	MGT	2001	A	3.67
18	84	NH1	ARG	1716	A	---	969	O2B	MGT	2001	A	2.71
19	85	NH2	ARG	1716	A	---	965	O3G	MGT	2001	A	2.98
20	85	NH2	ARG	1716	A	---	968	O1B	MGT	2001	A	3.42
21	85	NH2	ARG	1716	A	---	969	O2B	MGT	2001	A	3.75
22	86	N	GLN	1717	A	---	989	O6	MGT	2001	A	2.75
23	87	CA	GLN	1717	A	---	989	O6	MGT	2001	A	3.6
24	90	CB	GLN	1717	A	---	989	O6	MGT	2001	A	3.36
25	90	CB	GLN	1717	A	---	990	N1	MGT	2001	A	3.84
26	91	CG	GLN	1717	A	---	989	O6	MGT	2001	A	3.73
27	91	CG	GLN	1717	A	---	990	N1	MGT	2001	A	3.74
28	92	CD	GLN	1717	A	---	990	N1	MGT	2001	A	3.56
29	93	OE1	GLN	1717	A	---	988	C6	MGT	2001	A	3.83
30	93	OE1	GLN	1717	A	---	990	N1	MGT	2001	A	2.67
31	93	OE1	GLN	1717	A	---	991	C2	MGT	2001	A	3.22
32	93	OE1	GLN	1717	A	---	992	N2	MGT	2001	A	2.95
33	178	CG	TYR	1728	A	---	990	N1	MGT	2001	A	3.66

34	178	CG	TYR	1728	A	---	991	C2	MGT	2001	A	3.69
35	179	CD1	TYR	1728	A	---	988	C6	MGT	2001	A	3.62
36	179	CD1	TYR	1728	A	---	990	N1	MGT	2001	A	3.73
37	180	CD2	TYR	1728	A	---	991	C2	MGT	2001	A	3.61
38	180	CD2	TYR	1728	A	---	993	N3	MGT	2001	A	3.63
39	181	CE1	TYR	1728	A	---	985	N7	MGT	2001	A	3.87
40	181	CE1	TYR	1728	A	---	987	C5	MGT	2001	A	3.54
41	181	CE1	TYR	1728	A	---	988	C6	MGT	2001	A	3.71
42	181	CE1	TYR	1728	A	---	994	C4	MGT	2001	A	3.88
43	182	CE2	TYR	1728	A	---	993	N3	MGT	2001	A	3.53
44	182	CE2	TYR	1728	A	---	994	C4	MGT	2001	A	3.61
45	183	CZ	TYR	1728	A	---	983	N9	MGT	2001	A	3.71
46	183	CZ	TYR	1728	A	---	987	C5	MGT	2001	A	3.64
47	183	CZ	TYR	1728	A	---	994	C4	MGT	2001	A	3.49
48	184	OH	TYR	1728	A	---	983	N9	MGT	2001	A	3.46
49	184	OH	TYR	1728	A	---	984	C8	MGT	2001	A	3.48
50	184	OH	TYR	1728	A	---	985	N7	MGT	2001	A	3.83
51	184	OH	TYR	1728	A	---	994	C4	MGT	2001	A	3.76
52	633	SE	MSE	1782	A	---	980	C2'	MGT	2001	A	3.66
53	633	SE	MSE	1782	A	---	981	O2'	MGT	2001	A	3.7
54	634	CE	MSE	1782	A	---	991	C2	MGT	2001	A	3.4
55	634	CE	MSE	1782	A	---	992	N2	MGT	2001	A	3.31
56	634	CE	MSE	1782	A	---	993	N3	MGT	2001	A	3.27

Number of hydrogen bonds: 5

Number of non-bonded contacts: 56
