# Supplementary material for: Structure of a functional cap-binding domain in Rift Valley fever virus L protein
Source: PLoS Pathog. 2019 May 28;15(5):e1007829. doi: 10.1371/journal.ppat.1007829 (PMC6555543; doi:10.1371/journal.ppat.1007829)
Supplement: S3 Table — (PDF) [file ppat.1007829.s003.pdf]

**S3 Table. Functional analysis of L protein mutants in the RVFV minireplicon system.**[1]

| | Renilla luciferase activity (sRLU) | | RNA expression level (Northern blot signal) | |
|---|---|---|---|---|
| Mutant | % of wild-type[2] | Signal-to-noise ratio[3] | Antigenome level % of wild-type[4] | mRNA-to-antigenome ratio, relative to wild-type[5] |
| F1713Y | 96 +/– 10 | 2434 | 89 | 0.80 |
| F1713W | 92 +/– 17 | 1976 | 170 | 0.54 |
| F1713H | <1 +/– <1 | 8 | 3 | 0.88 |
| F1713A | <1 +/– <1 | 9 | 11 | 0.28 |
| Y1728F | 99 +/– 25 | 2250 | 118 | 0.44 |
| Y1728W | 153 +/– 29 | 3807 | 62 | 1.12 |
| **Y1728H** | **42 +/– 9** | **859** | **112** | **0.14** |
| **Y1728E** | **5 +/– 1** | **102** | **110** | **0.07** |
| **Y1728A** | **6 +/– <1** | **127** | **122** | **0.05** |
| R1716K | 127 +/– 21 | 3213 | 120 | 0.70 |
| **R1716E** | **9 +/– 3** | **194** | **221** | **0.04** |
| **R1716M** | **25 +/– 3** | **560** | **238** | **0.07** |
| **R1716A** | **54 +/– 20** | **1187** | **220** | **0.25** |
| **Q1717K** | **4 +/– <1** | **86** | **63** | **0.12** |
| **Q1717A** | **50 +/– 10** | **1019** | **199** | **0.13** |
| I1747V | 156 +/– 15 | 3930 | 127 | 0.86 |
| I1747A | 14 +/– 11 | 375 | 43 | 0.35 |
| I1747T | 34 +/– 17 | 831 | 112 | 0.31 |
| **N1749D** | **8 +/– 2** | **145** | **44** | **0.23** |
| N1749T | 105 +/– 15 | 2637 | 238 | 0.62 |
| N1749L | <1 +/– <1 | 21 | 17 | 0.30 |
| N1749A | 84 +/– 28 | 1638 | 110 | 0.62 |
| M1782H | <1 +/– <1 | 17 | 6 | 0.97 |
| M1782Q | 21 +/– 8 | 475 | 81 | 0.49 |
| M1782L | 23 +/– 15 | 569 | 44 | 1.13 |

| | | | | | | |
|---|---|---|---|---|---|---|
| M1782I | 32 | +/– | 7 | 791 | 20 | 1.64 |
| M1782A | <1 | +/– | <1 | 26 | 12 | 0.88 |
| W1778F | 66 | +/– | 10 | 1653 | 78 | 0.74 |
| W1778A | <1 | +/– | <1 | 1 | 5 | 0.88 |
| W1778L | 2 | +/– | 1 | 55 | 12 | 0.91 |

[1] For each mutant, three independent transfection experiments were performed. Renilla luciferase values represent mean with standard deviation (n = 3). Northern blots were performed once per mutant. A selective defect in mRNA synthesis was defined as reduction in Renilla luciferase level (<1–55%) despite wild-type like antigenome synthesis (40–240%) and reduction of the mRNA-to-antigenome ratio (1–25%). Mutants with a selective defect in mRNA synthesis are shown in boldface on grey background.

[2] Standardized relative light unit (sRLU) value (wild-type L protein = 100%).

[3] sRLU value of mutant divided by sRLU value of negative control mutant containing a mutation in the catalytic site of the RNA-dependent RNA polymerase.

[4] Antigenome signals in Northern blots were quantified via intensity profiles using ImageJ2 software (wild-type L protein = 100%).

[5] RNA signals in Northern blots were quantified using ImageJ2 software and the mRNA-to-antigenome signal ratio was calculated. The wild-type ratio was set at 1 for each experiment (i.e., the signal ratio of a mutant was normalized with the wild-type ratio) to render independent experiments comparable.
