# Supplementary figures and images for: Structure of a functional cap-binding domain in Rift Valley fever virus L protein
Source: PLoS Pathog. 2019 May 28;15(5):e1007829. doi: 10.1371/journal.ppat.1007829 (PMC6555543; doi:10.1371/journal.ppat.1007829)

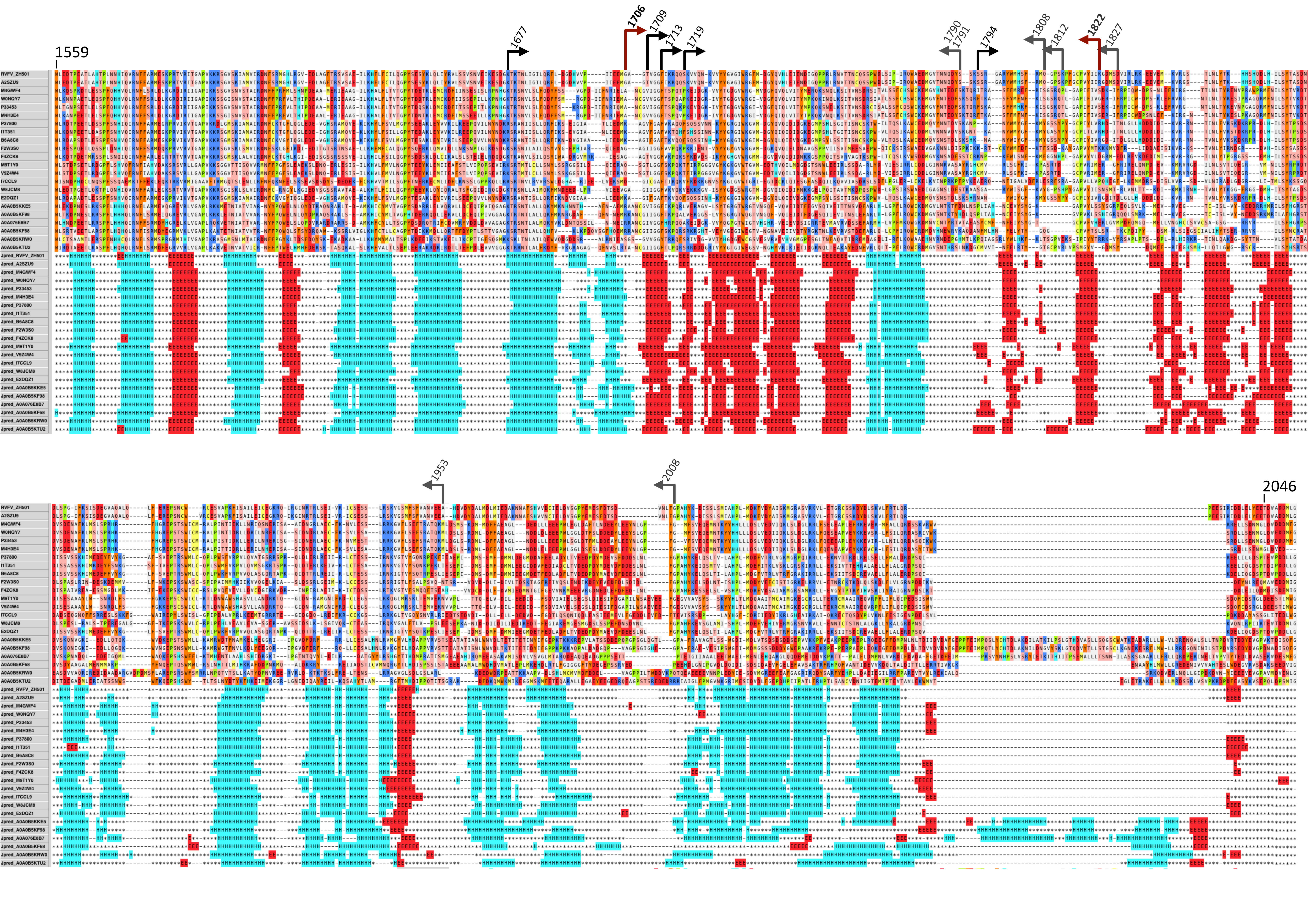

Supplement: S1 Fig — The figure presents the secondary-structure based alignment of the L protein C-terminal sequences of 22 phlebo- and banyangviruses (Uniprot accession numbers are given). Chemically similar residues are depicted in the same color. The corresponding secondary structure prediction was calculated by Jpred4 [60] and is depicted below the sequences (β-sheets in red, α-helices in blue, asterisks for loops). N- and C-terminal boundaries of tested constructs are indicated by arrows above the sequences. N- and C-termini of RVFV CBD13 are indicated with red arrows. All numbers refer to RVFV strain ZH-501 full-length L protein. (TIF) [file ppat.1007829.s004.tif]

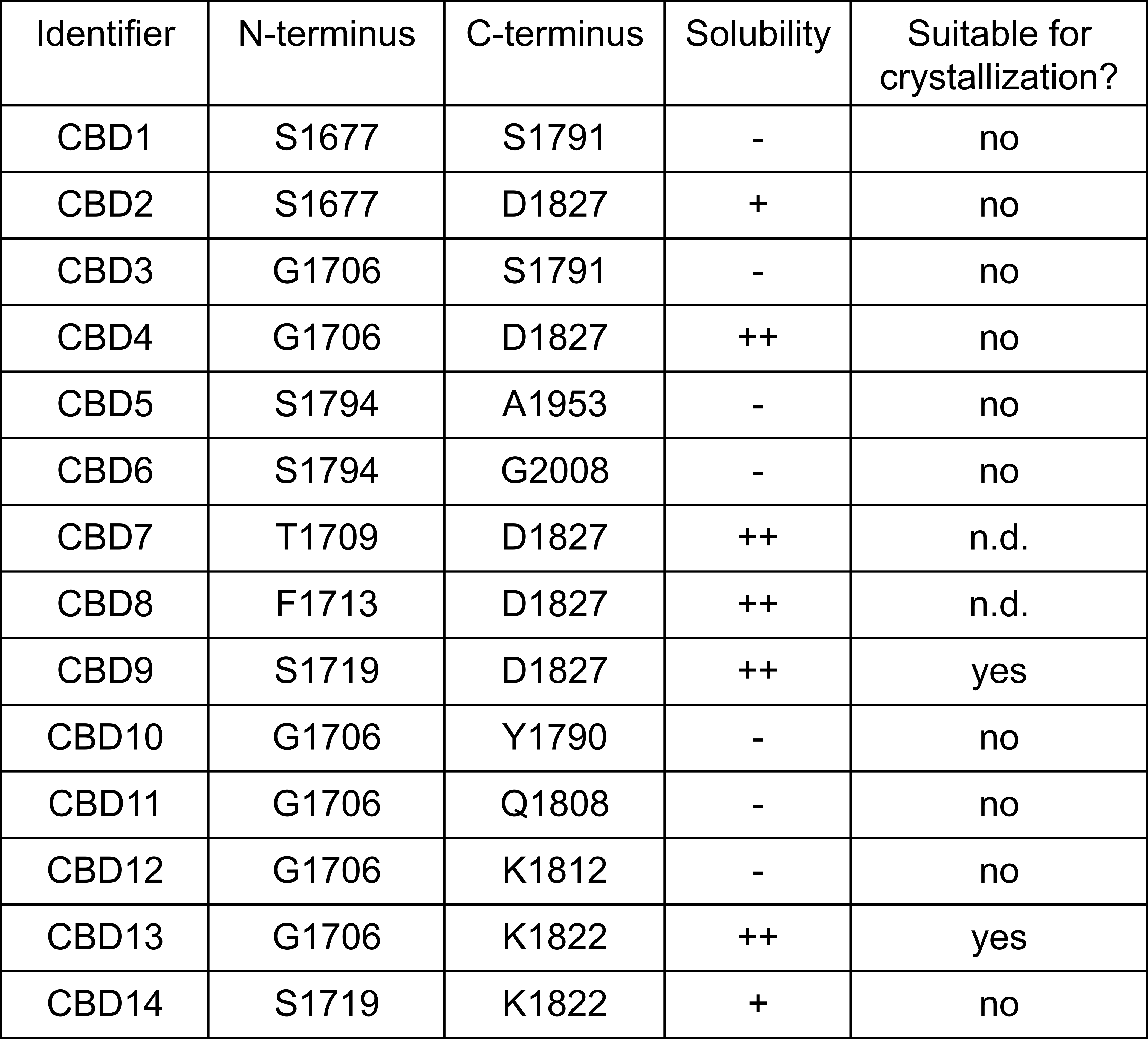

Supplement: S2 Fig — An overview of all RVFV constructs designed and tested is given: the respective N- and C-termini, rating of protein solubility from insoluble (-) and somehow soluble (+) up to highly soluble (++), as well as information about suitability for crystallization (no, yes, n.d. = not determined). (TIF) [file ppat.1007829.s005.tif]

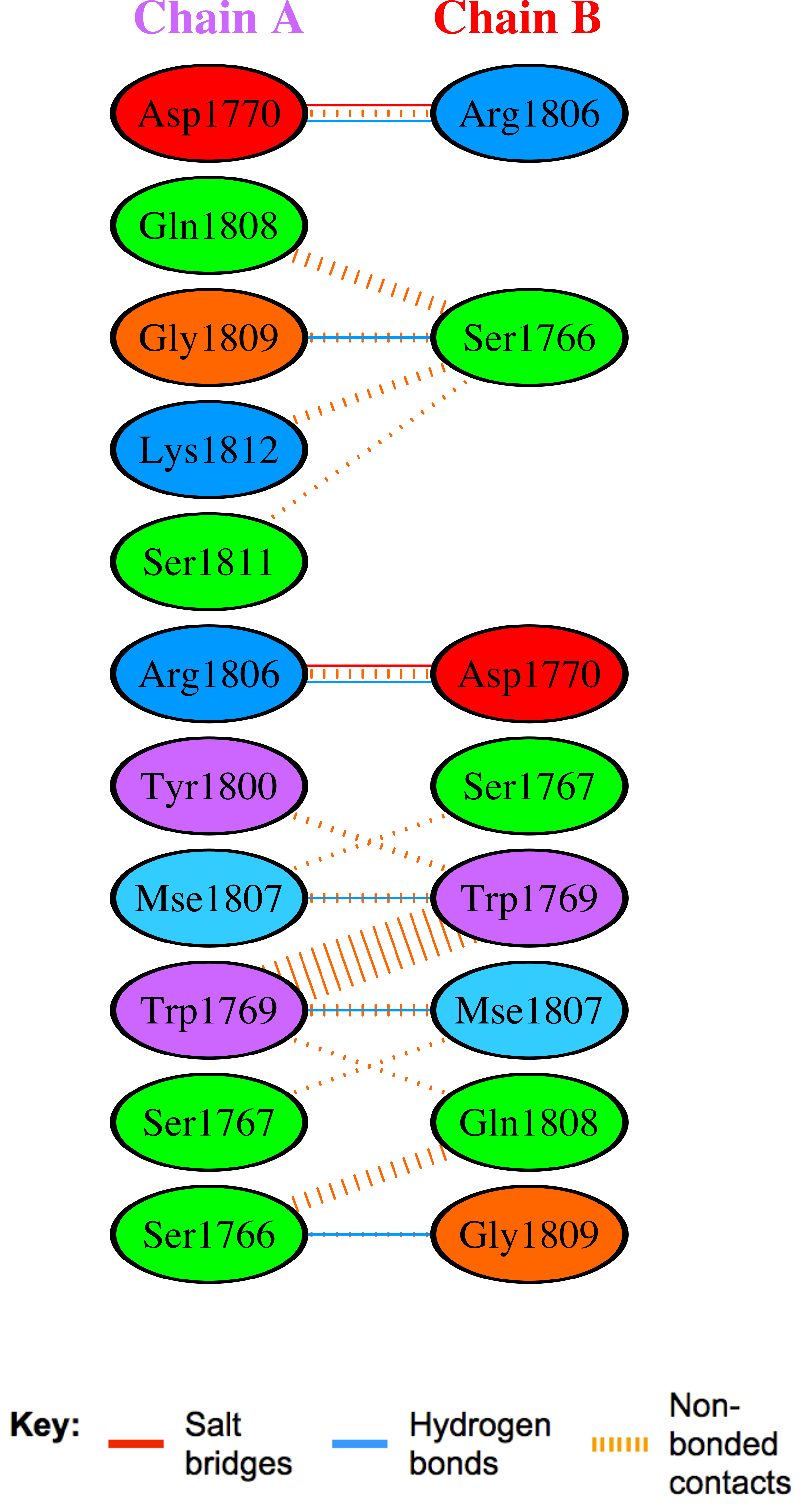

Supplement: S3 Fig — The figure was taken from PDBsum [59] to summarize the interactions observed between the two monomers in the crystal structure. (TIF) [file ppat.1007829.s006.tif]

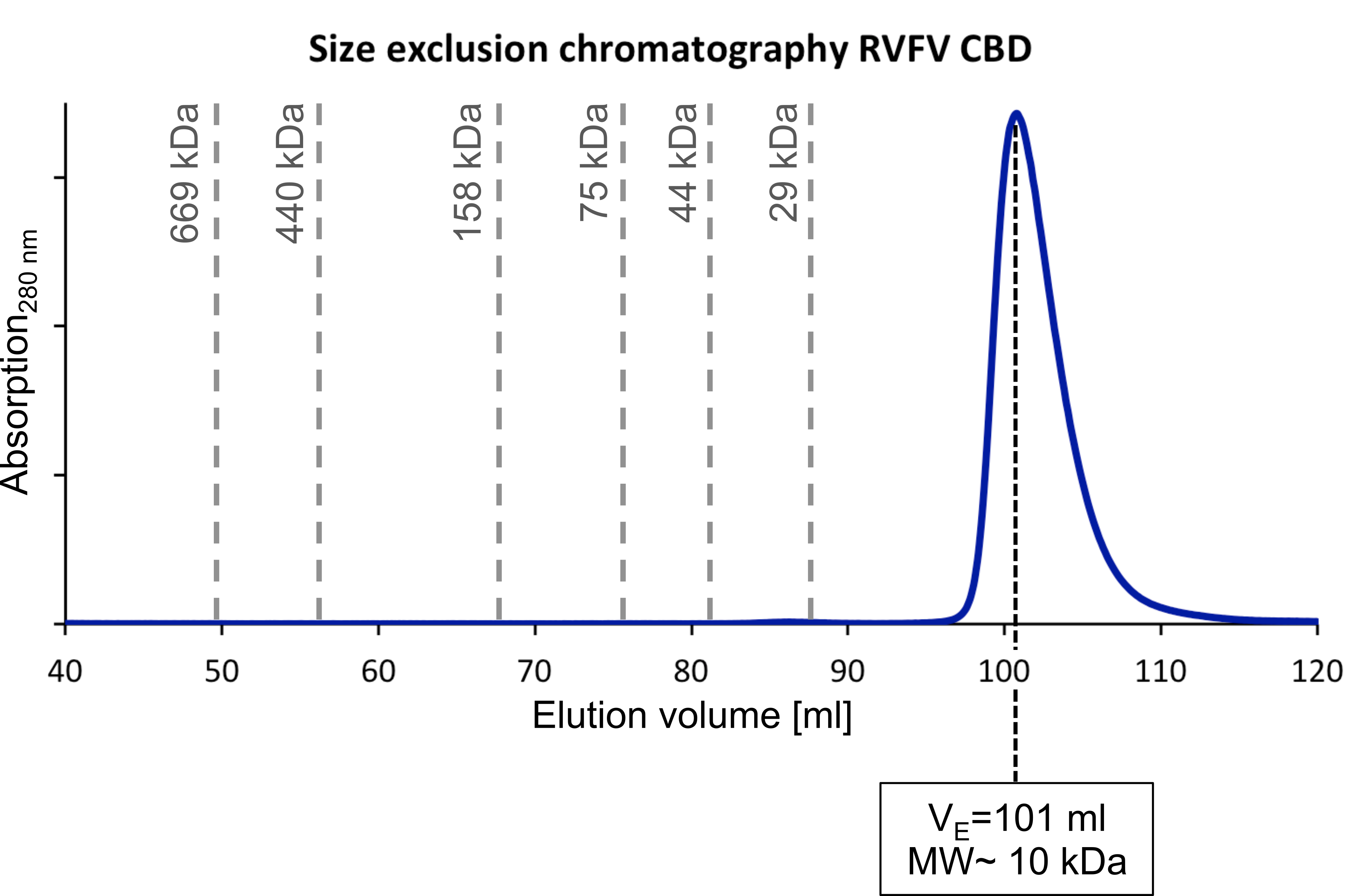

Supplement: S4 Fig — An example of a size-exclusion chromatogram is given with the absorption at wavelength 280 nm being monitored. Chromatography was performed on a Superdex 200 16/60 HiLoad column at 4°C with a buffer containing 50 mM Na-phosphate, 100 mM NaCl and 10% glycerol. Elution volumes of calibration proteins are indicated by dotted lines and labelled with the respective molecular weight of the protein. The estimated molecular weight of the protein in the peak fraction was calculated based on the column calibration and is displayed below the graph. (TIF) [file ppat.1007829.s007.tif]

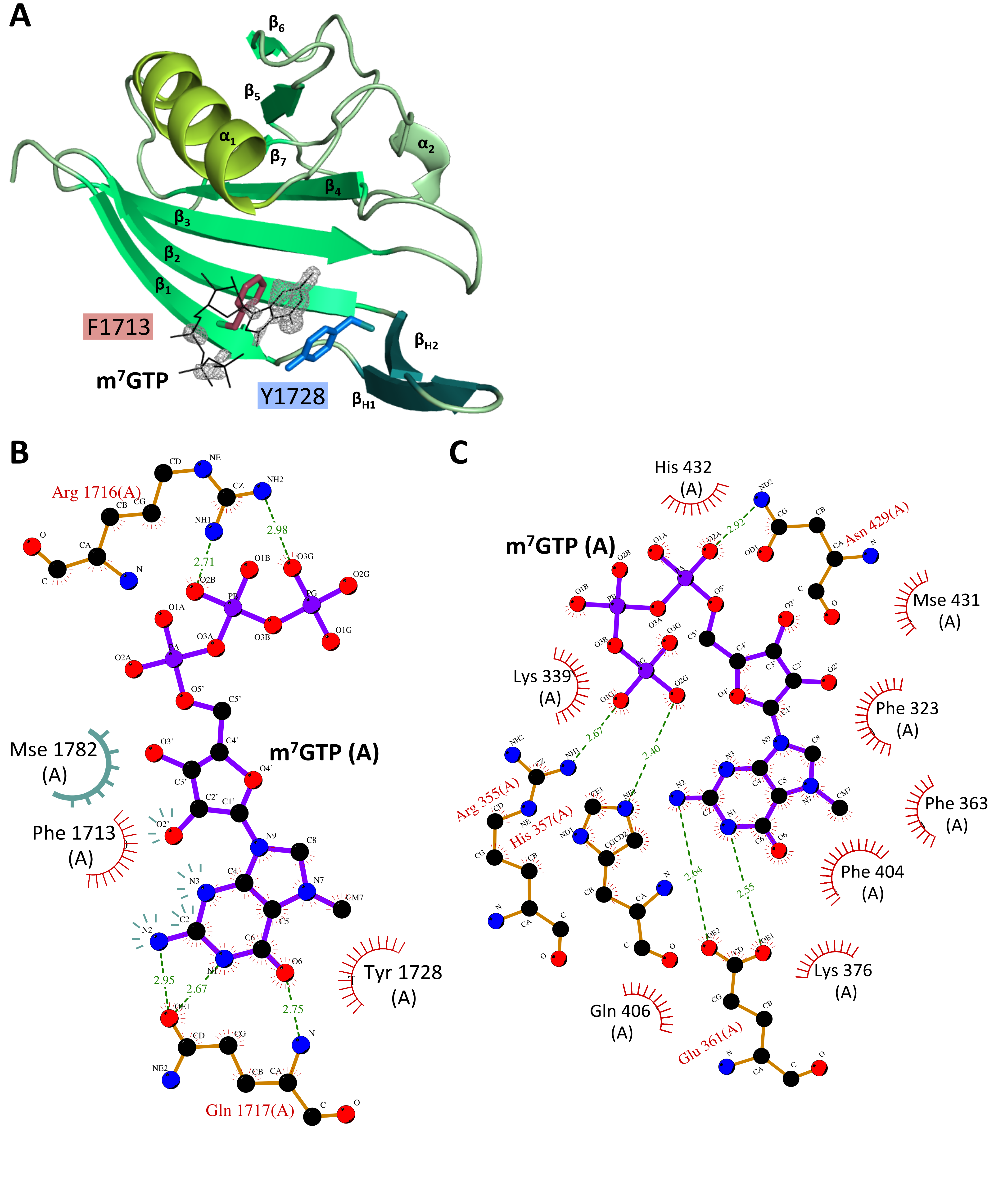

Supplement: S5 Fig — A) The figure shows binding of m7GTP to CBD13 chain B in the crystal. CBD13 is presented as a ribbon diagram with the side chains of the two aromatic residues typical for binding of cap-structures shown as sticks. m7GTP is presented as lines and the surrounding electron density (2|Fo|-|Fc| omit map at 1.5σ) as grey mesh. Secondary structure elements are labelled. B) A ligand plot for interaction of m7GTP with RVFV CBD chain A is presented. The plot was generated by PDBsum [59] and modified to also include M1782. A detailed list of interactions is displayed in S2 Table C) A ligand plot for interaction of m7GTP with influenza virus PB2 (PDB:2VQZ, chain A) is presented. The plot was generated by PDBsum [59] and modified for presentation reasons. (TIF) [file ppat.1007829.s008.tif]

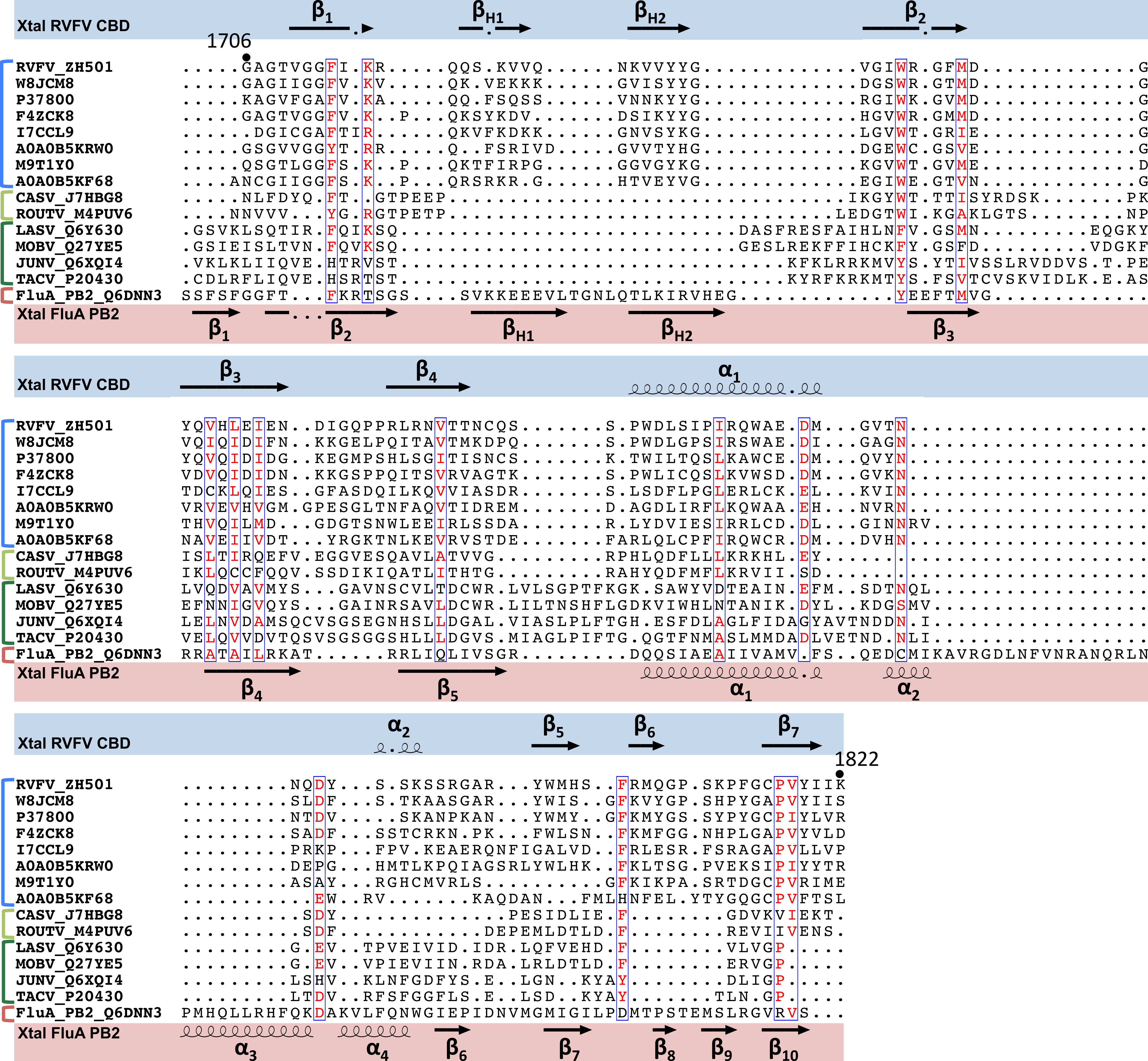

Supplement: S6 Fig — This figure presents an alignment of the (putative) cap-binding domains of 2 reptarenaviruses (light green label), 4 mammarenaviruses (dark green label), 7 phleboviruses and 1 banyangvirus (blue label) and influenza A virus (red label). The alignment was essentially based on predicted secondary structures and calculated using PRALINE software [53, 56] but required major manual adjustments. Graphical presentation of the alignment was done using ESPript (http://espript.ibcp.fr) [57]. The secondary structure of RVFV CBD (PDB 6QHG, on blue background) and influenza virus cap-binding domain (PDB 2VQZ, on red background) are displayed above and below the sequences, respectively. Secondary structure elements are labelled according to Fig 6. Numbering refers to RVFV strain ZH-501 full-length L protein. (TIF) [file ppat.1007829.s009.tif]

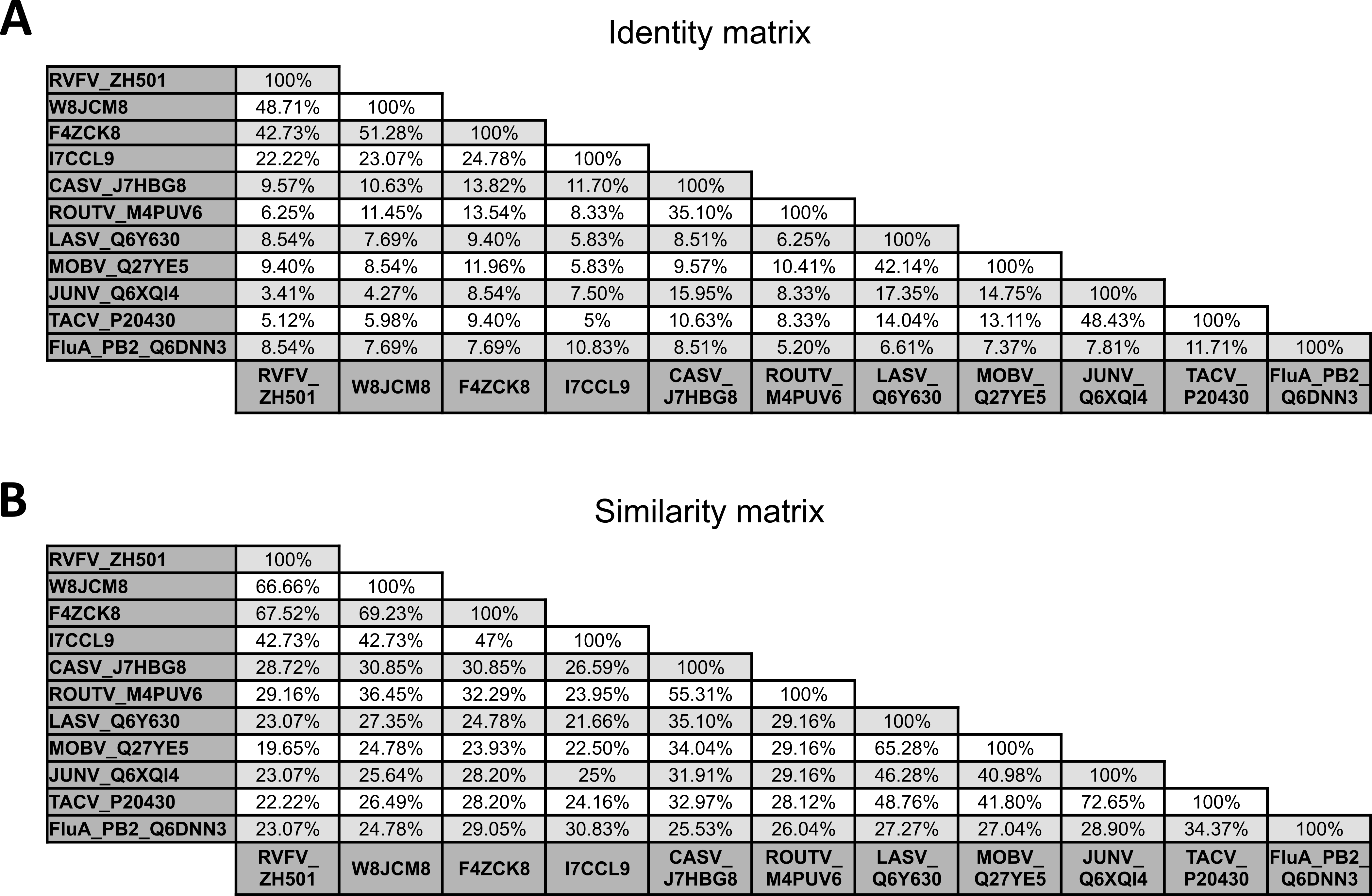

Supplement: S7 Fig — Identity and similarity matrices calculated by SIAS online tool (http://imed.med.ucm.es/Tools/sias.html) are presented for selected sequences of the alignment displayed in S6 Fig. (TIF) [file ppat.1007829.s010.tif]

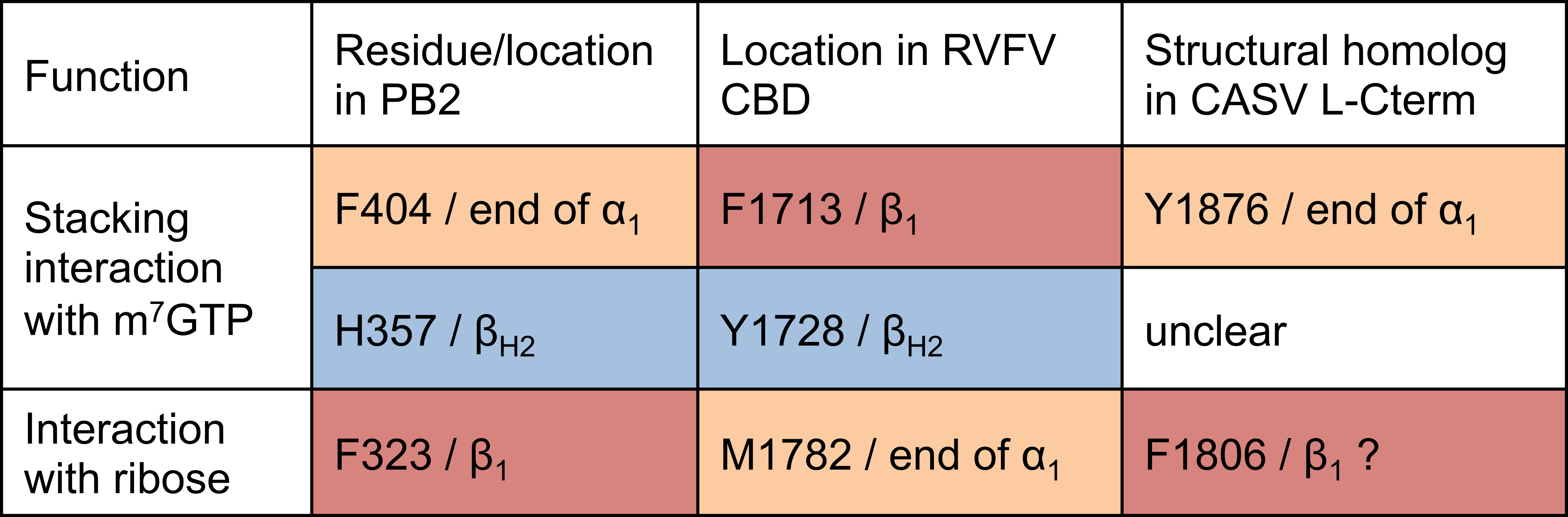

Supplement: S8 Fig — The amino acids (putatively) interacting with an m7GTP ligand are compared between the structures of CASV L-Cterm (5MUZ), RVFV CBD (6QHG) and influenza virus PB2 (2VQZ) according to Fig 6. The location of the residues is given and structurally corresponding residues are marked with the same color. As for the CASV putative cap-binding domain no m7GTP binding has been demonstrated, the displayed residues solely correspond to structural homologs. (TIF) [file ppat.1007829.s011.tif]

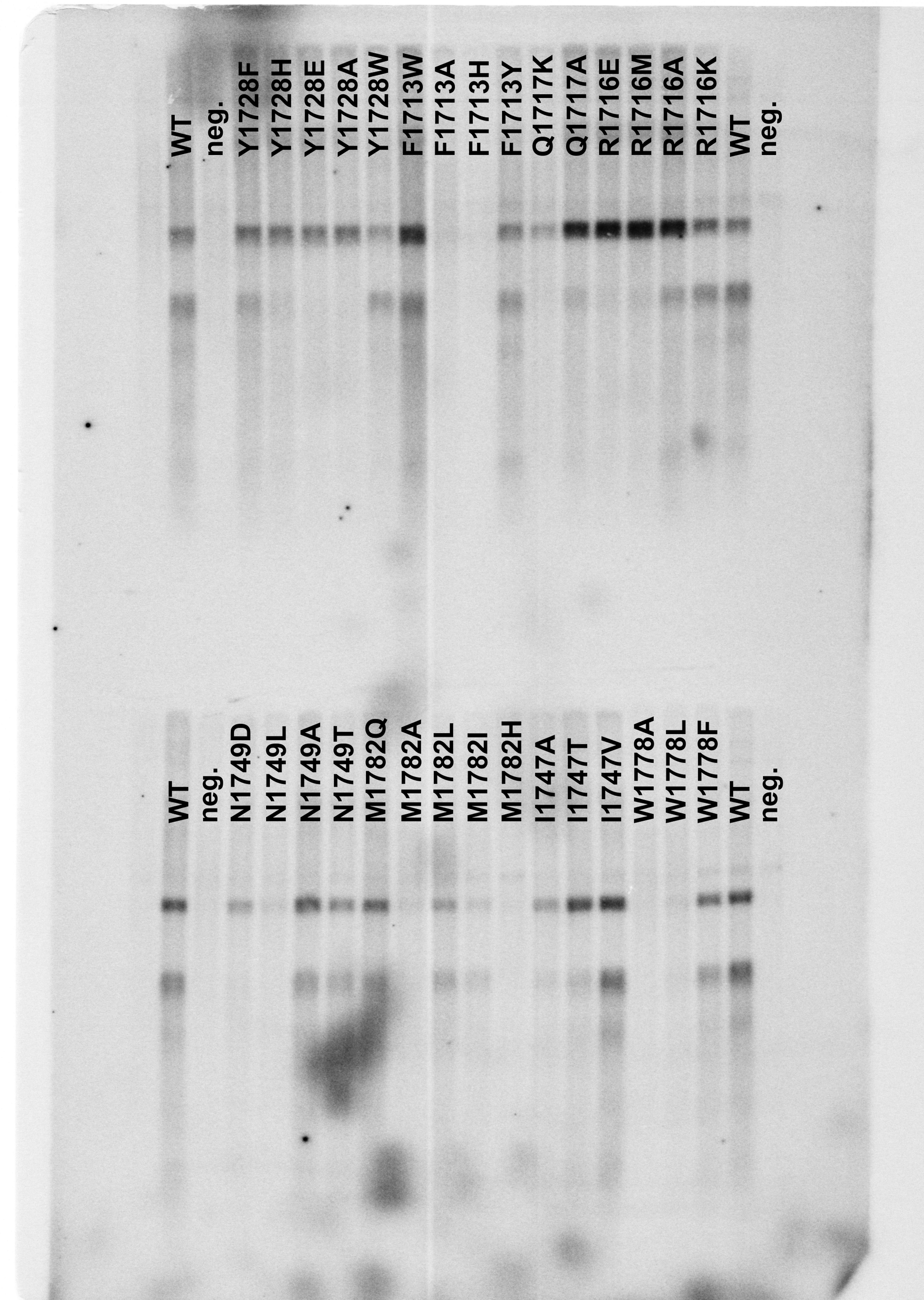

Supplement: S9 Fig — This figure provides the original northern blot autoradiogram presented in Fig 5. (TIF) [file ppat.1007829.s012.tif]
